# Smart Egg Processing in the Digital Food Era, Part I: Advanced Sensing and Artificial Intelligence for Non-Destructive Egg Quality and Safety Assessment

**DOI:** 10.3390/foods15213956

**Published:** 2026-11-04

**Authors:** Osama K. Abou-Emera, Mohammed Aladhadh

**Affiliations:** 1 Department of Animal and Poultry Production, College of Agriculture and Food, Qassim University, Buraydah 51452, Saudi Arabia; 2 Department of Poultry Breeding Research, Animal Production Research Institute, Agriculture Research Centre, Dokki, Giza 12618, Egypt; 3 Department of Food Science and Human Nutrition, College of Agriculture and Food, Qassim University, Buraydah 51452, Saudi Arabia; aladhadh@qu.edu.sa

**Keywords:** egg quality, non-destructive sensing, hyperspectral imaging, machine learning, deep learning, food safety, authentication, Industry 4.0

## Abstract

Eggs are still frequently evaluated through periodic destructive tests, while high-throughput processing increasingly requires non-destructive measurements that can support timely decisions. This Part I critical review examines the sensing and prediction layer of smart egg processing, covering computer vision, hyperspectral and multispectral imaging, near-infrared, Raman and fluorescence spectroscopy, electronic-nose sensing, biosensors, and machine-learning methods used to interpret these signals. The evidence shows that no single modality provides complete coverage of external, internal, chemical, and microbiological attributes and that multimodal fusion is no longer merely conceptual: egg-specific studies have demonstrated the fusion of spectral and olfactory information and, more recently, image, shape, and weight features. Explainable AI has likewise entered egg research, including the SHAP-based interpretation of NIR and hyperspectral models. The more persistent limitation is the weakness and inconsistency of validation beyond the development dataset. Small or single-source datasets, incomplete reporting of preprocessing and feature-selection procedures, possible data leakage, class imbalance, limited calibration and uncertainty analysis, and scarce external or prospective validation constrain claims of industrial readiness. The review consequently reframes the central research gap as trustworthy generalization: demonstrating that sensing and AI remain accurate, interpretable, and decision-useful under the biological, environmental, and operational variability of real egg-processing systems.

## 1. Introduction

By 2024, global egg production had reached an estimated 100 million tonnes, with hen eggs alone contributing 94% of this output [1], making the sector one of the fastest-growing categories of animal protein over the past decade [2]. This expansion has not been uniform. It has been driven mainly by large, vertically integrated operations engineered to move eggs from lay to pack within hours—with throughput rates at which the visual or manual inspection of every egg is impractical. Set against this pace of industrial change, quality-assurance practices have changed little. Candling, manual grading, and periodic destructive sampling for the Haugh unit, yolk index, or microbial load remain widely used on processing lines. These methods are not without merit, but they have three limitations that become more important as production speeds increase: they examine only a small subsample of output rather than every unit, they destroy the very eggs that they evaluate, and, most importantly, they generate quality information after the fact, at a point when corrective action is no longer possible.

Two technological developments could help to close this gap. The first is the maturing field of non-destructive sensing—spanning optical, spectroscopic, and chemical modalities—capable of characterizing an egg’s internal and external state without compromising shell integrity or removing units from the line. The second is the parallel advance of machine-learning and deep-learning architectures able to transform the resulting high-dimensional outputs, be it a hyperspectral cube, a volatile organic compound fingerprint, or a spectral curve, into actionable predictions of quality or safety within a near real-time window. Neither development is novel on its own: the spectroscopic assessment of egg freshness has been studied for decades [3], and the computer-vision detection of shell cracks was reported as early as 1991 [4]. The newer idea, as well as the motivation for this review, is to couple these sensing and AI capabilities directly to processing decisions, so that a measured or predictEgg quality and safety attributes relevant toEgg quality and safety attributes relevant toed attribute does not simply flag a defective egg for rejection but actively steers which processing route, thermal or non-thermal, mild or intensive, an egg or a batch of liquid egg receives. This coupling is prospective. The studies reviewed in Part I demonstrate sensing and prediction. No experimentally demonstrated case of sensor-guided selection of a food-safety intervention was identified in the material examined, so statements about such decisions in this review describe a proposed architecture, not an existing system.

**Figure 1:**
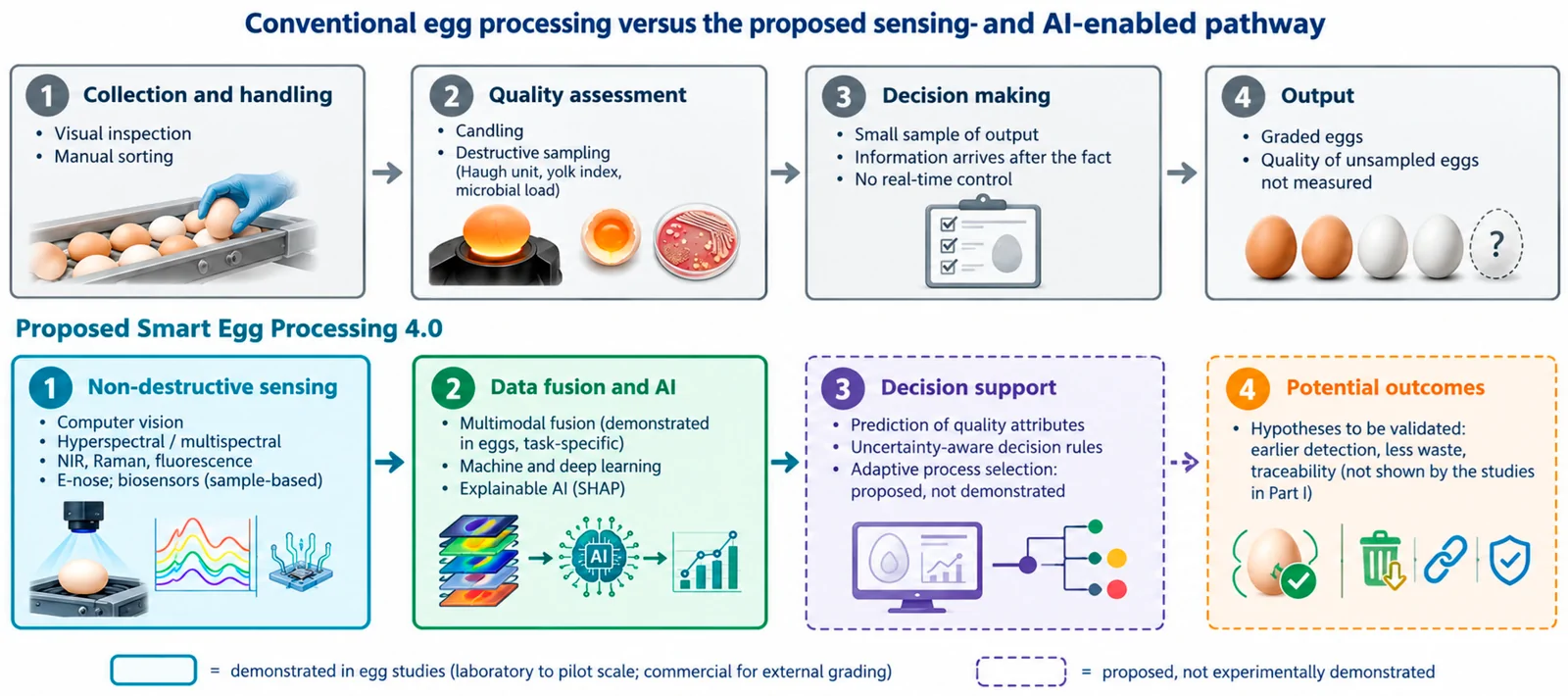
Evolution from conventional egg processing to Smart Egg Processing 4.0. The upper row summarizes conventional egg processing, characterized by manual or visual inspection, periodic assessment, fixed processing conditions, and end-product inspection. The lower row illustrates the corresponding smart-processing capabilities enabled by non-destructive sensing, AI/ML-based prediction, and emerging adaptive and non-thermal processing approaches. Solid outlines mark steps demonstrated in egg studies; dashed outlines mark steps that are proposed and have not been experimentally demonstrated. Sensing and AI-enabled assessment are more mature than adaptive, batch-specific process selection. The potential outcomes shown are hypotheses to be validated, not results of the studies reviewed.

We define this convergence as smart egg processing—a system in which advanced sensing, artificial intelligence and machine learning, and non-thermal or minimally invasive processing technologies are coordinated through continuous, real-time data flows, enabling quality grading, safety verification, and process optimization to proceed concurrently rather than as discrete, sequential stages. It differs from conventional egg processing in three respects. First, measurement is continuous and non-destructive rather than periodic and destructive. Second, the processing intensity applied to a batch can in principle be informed by what the sensors and models report about that specific batch, rather than fixed by a single validated thermal schedule applied uniformly regardless of the incoming material’s condition. Third, functional and nutritional preservation is treated as a design objective on equal footing with microbial safety, not as an afterthought to be checked once a pasteurization process has already been fixed. The conceptual transition from conventional egg processing toward a sensing- and AI-enabled Smart Egg Processing 4.0 framework is summarized in Figure 1.

The pressure to preserve functional and nutritional quality is not incidental. Eggs owe much of their commercial value in bakery, confectionery, and prepared-food applications to protein-driven functional properties, foaming, gelling, and emulsifying, which are highly sensitive to thermal denaturation [5]. Standard egg pasteurization (57 °C for approximately 57.5 min for shell eggs in some regulatory schemes) inactivates Salmonella effectively but at a cost to those functional properties and to heat-labile micronutrients, and coverage remains partial: in the United States, for instance, well under 3% of shell eggs are pasteurized under the traditional thermal schedule [6]. This tension, between doing enough to the egg to make it safe and doing too much to the egg to keep it useful, is where non-thermal technologies (ultraviolet and pulsed light, cold plasma, high hydrostatic pressure, pulsed electric fields, ultrasound, and novel thermal alternatives such as radiofrequency heating) have attracted sustained research interest over the last fifteen years [5,6]. This review asks whether AI-guided sensing can close that gap: identifying which eggs warrant aggressive treatment and which do not, and tuning process parameters to the minimum intensity needed to meet a given safety target. Existing reviews do not fully answer this question, because each focuses on one technology family. Sensing-focused reviews—most comprehensively Ahmed and colleagues’ synthesis of optical sensing technologies for “Egg Industry 4.0” [7], along with three more recent treatments devoted specifically to freshness-detection technologies [8,9,10]—cover sensing and prediction in depth but do not address processing decisions. Processing-focused reviews cover non-thermal pasteurization technologies [6] and processing-driven changes in egg protein functionality [5], but neither addresses AI-based sensing or process control substantively. Sensing and prediction on one side and processing and preservation on the other have therefore each been reviewed repeatedly, while the connection between them has received little attention. Table 1 compares the six closest reviews and states what the present review adds to each.

Two points follow from this comparison. First, none of the earlier reviews is superseded by the present one; each remains the more detailed reference for its own technology family, and this review uses them as foundations rather than attempting to reproduce their depth. Second, the central gap claimed here should not be framed as the absence of multimodal sensing or explainable AI. Egg-specific multimodal fusion has now been demonstrated, and SHAP-based interpretation has been reported for egg NIR and hyperspectral models. The more defensible gap is the lack of a mature evidence chain connecting heterogeneous sensing, calibrated and interpretable prediction, uncertainty-aware decision rules, and independently validated process actions. The novelty claimed by this review is therefore critical and architectural: it evaluates how trustworthy the sensing-to-decision pathway is, rather than presenting multimodality or AI itself as new.

Given the breadth of evidence that this synthesis requires, spanning multiple sensing modalities, AI/ML paradigms, and the systems-level questions of validation, real-time control, traceability, sustainability, and industrial scale-up, this review is presented in two parts. This paper, Part I, addresses the sensing and prediction half of the smart egg-processing pathway: Section 2 defines the quality and safety attributes a smart system needs to measure or predict and distinguishes direct measurement from statistical inference; Section 3 evaluates the sensing technologies; Section 4 examines AI/ML methods, explainability, and validation; and Section 5 reviews application-specific prediction tasks. Part II takes up non-thermal processing, functional and nutritional preservation, real-time control, digital traceability, sustainability, and industrial implementation. Across both parts, evidence is classified by validation tier wherever the underlying literature permits, and evidence from non-egg food systems is not treated as direct egg evidence. Part I does not rely on Part II to support any of its conclusions about sensing and prediction. Technologies for which the egg-specific literature is thin (mid-infrared, terahertz, ultrasound, dielectric, thermal, X-ray, and low-field NMR methods) are treated briefly in Section 3.4 and Section 3.9, and the coverage of these methods is not claimed to be complete.

## 2. Egg Quality and Safety Attributes Relevant to Smart Processing

This manuscript is a critical narrative review rather than a formal systematic review or meta-analysis, and it does not report a protocol-driven systematic search for the original selection of sources. Accordingly, the purpose of the evidence framework is not to assign pooled effect sizes, but to distinguish what has been demonstrated from what remains plausible. Studies are interpreted according to five questions: (i) what attribute is measured or predicted; (ii) whether the signal is direct or a proxy; (iii) whether preprocessing, feature selection, and model tuning were isolated from the test data; (iv) whether validation is internal, held-out same-source, independent-source, cross-facility, cross-season/geographic, or prospective production-line validation; and (v) whether uncertainty, class imbalance, and operational decision costs are reported. The terms “not reported” and “not demonstrated” are therefore used deliberately: the absence of evidence in a paper is not treated as evidence that a procedure was not performed. This distinction becomes particularly important when comparing apparently high accuracies across studies with different datasets and validation designs.

Supplementary literature search. The evidence base was checked in two steps. First, targeted queries were run on a general web search engine on 23 and 24 October 2026 (queries and outcomes: Supplementary Table S1). These covered acoustic crack detection, the Raman authentication of rearing systems, the surface plasmon resonance detection of egg-yolk antibodies, wavelength selection in near-infrared egg studies, terahertz and ultrasound methods, commercial grading lines, eggshell porphyrin fluorescence, and hen age in shelf-life models. Second, structured searches were run on 25 October 2026 in Scopus, Europe PMC, PubMed, Lens.org, and Google Scholar, combining an egg term with a non-destructive term and with terms for the target (freshness, cracks, defects, authentication, Salmonella), the sensing method and the analysis method (query strings and result counts: Supplementary Table S1). Web of Science was not searched. The five database searches returned 112 records (Scopus 66, Europe PMC 14, PubMed 11, Lens.org 1, Google Scholar 20, the latter being the first results page of two queries); 81 remained after the removal of 31 duplicates. Titles and abstracts (titles only for Google Scholar) were screened by the authors, assisted by an AI tool. Claude Sonnet 5.5 (Anthropic) assisted with the supplementary literature search, with the screening of the retrieved records and with reference verification; in the revised version, it also reconciled the screening counts, extracted sample sizes, and validation designs from the full texts supplied by the authors and drafted the corresponding text and tables. All AI outputs were cross-checked against the source documents and reviewed by the authors, who take full responsibility for the content. Of the 81 unique records, 18 were already cited. Of the remaining 63, 20 were excluded as outside the scope (hatching-egg fertility or sexing, egg products or other species, records not specific to eggs, or front matter), 1 on chemical residues was set aside, 10 conference, book-chapter, or Chinese-language records were not assessed, and 32 were retained as candidates; 12 of these were added to the review [12,13,14,15,16,17,18,19,20,21,22,23] after their full texts, supplied by the authors, had been read; 1 more was read and excluded because it is not specific to eggs; the other 19 were not read in full, are not cited, and have no final inclusion decision, so the conclusions of this review rest on the sources cited here and the effect of those 19 was not assessed (Supplementary Table S6). Apart from these 13 studies, no full-text screening or formal quality appraisal was performed. A source was added only when it was peer-reviewed or an institutional record, specific to eggs, and directly supported a statement and when its bibliographic record could be confirmed from the publisher, journal, repository, or a citing article.

Evidence tiers. To keep the validation language consistent across sections and tables, the following definitions are used. Tier 1: training or resubstitution performance only. Tier 2: internal cross-validation, including nested cross-validation, on the development dataset. Tier 3: a held-out test set drawn from the same acquisition campaign (same flock, facility, instrument, and period) as the training data. Tier 4: independent-source validation, meaning new eggs obtained separately from the development set, for example from a different flock or lot or bought at retail, but measured with the same laboratory workflow. Tier 5: cross-facility, cross-season, or geographic validation, in which the site, instrument, season, or population changes. Tier 6: prospective validation on a running production line. Some papers call everything from Tier 3 upward “external”; here, only Tiers 4 to 6 count as external, and only Tiers 5 and 6 test transfer between facilities or operating conditions. Each study is assigned the highest tier that its reported design clearly supports. Where the source does not describe how test data were separated from training data, the tier is recorded as not determinable and is not assumed.

Before sensing or AI architectures can be evaluated, the attributes that they are meant to measure must be defined. Egg quality and safety are not single, unitary variables but a bundle of external, internal, chemical, and microbiological attributes—some accessible to direct physical measurement, others obtainable only as a prediction from a correlated, indirect signal. Conflating these two categories is a recurring weakness in the sensing literature, and the distinction matters for a review of this kind: a model reported to “detect” *Salmonella* with 95% accuracy is making a different claim depending on whether it measured a bacterial marker directly or inferred contamination risk from an unrelated optical proxy such as shell color or cuticle integrity.

### 2.1. External Quality Attributes

Externally, egg quality is defined by a cluster of related attributes: shell integrity—including cracks, hairline fractures, and pinholes—shell color and its uniformity, overall shape and size, surface contamination from fecal material, blood, or dirt, and the condition of the cuticle. These features matter for two reasons. Cracks and pinholes compromise the shell’s role as a physical and antimicrobial barrier: eggshell penetration by Salmonella and other bacteria is strongly associated with cuticle loss and shell damage, because the cuticle occludes the pores through which bacteria would otherwise access the shell membranes [24]. Shape, size, and surface cleanliness, by contrast, are primarily grading and marketing attributes rather than safety attributes, though heavily soiled shells do increase the microbial load that any downstream washing or disinfection step must overcome. Nearly all external attributes are, in principle, directly measurable via optical means (machine vision, structured light, or acoustic-impulse response), which is why computer vision has historically been the first sensing technology adopted on commercial grading lines [7,25].

### 2.2. Internal Quality Attributes

Internal quality is where non-destructive assessment becomes difficult, because the parameters of interest have traditionally required breaking the egg. The Haugh unit, still the industry-standard freshness index nearly ninety years after its introduction [26], is calculated from albumen height and egg weight and requires the egg to be broken onto a flat surface. The yolk index (yolk height divided by yolk diameter), yolk color, air cell size, albumen pH, and the presence of blood or meat spots are similarly assessed destructively in conventional laboratory practice. Karoui and colleagues’ review of egg-freshness evaluation methods lays out how these classical destructive indices relate to the underlying biochemistry of storage-related deterioration: as an egg ages, carbon dioxide and moisture diffuse out through the shell, the albumen pH rises from roughly 7.6 toward 9 or higher, the thick albumen liquefies (thinning), and the yolk membrane weakens and allows water to migrate from the albumen to yolk, increasing the yolk size and flattening the yolk index [3]. Every non-destructive sensing technology discussed in Section 3 is, in effect, attempting to detect some optical, spectral, or dielectric correlate of these underlying biochemical changes without physically accessing them.

### 2.3. Chemical and Nutritional Characteristics

A third tier of attributes concerns the egg’s chemical composition: protein content and the relative proportions of ovalbumin, ovotransferrin, and other albumen proteins; the lipid content and fatty acid profile, concentrated almost entirely in the yolk; the moisture content; pH; the lipid and protein oxidation status; fat-soluble vitamins (A, D, E) and carotenoids (lutein, zeaxanthin), which are responsible for yolk color and carry documented antioxidant and eye-health relevance [27]; and other bioactive compounds such as phosvitin and immunoglobulin Y. These are generally the hardest attributes to access non-destructively, because they require spectroscopic techniques sensitive enough to resolve overlapping chemical signatures through an intact shell and because processing itself can alter them, a concern developed further in Part II.

### 2.4. Safety Characteristics

Safety-relevant attributes include microbial contamination (principally Salmonella Enteritidis and Salmonella Typhimurium, the serovars most consistently implicated in shell-egg and liquid-egg outbreaks [6]), indicators of spoilage such as total volatile basic nitrogen, chemical contaminants, and adulteration or authenticity (for example, mislabeling of production system, such as caged versus free-range or organic, which has motivated dedicated authentication research [28,29,30]). It is on this tier of attributes that the distinction between measurement and prediction is most consequential. No optical or spectroscopic sensor detects Salmonella cells directly under commercial conditions; what such sensors detect are proxies, volatile metabolites associated with bacterial activity, or physical damage (cracks) statistically associated with elevated contamination risk, or spectral shifts associated with the chemical byproducts of spoilage rather than with the causative organism itself. A prediction built on such a proxy is only as reliable as the correlation between the proxy and the underlying hazard, and that correlation can be disrupted by variables (hen strain, diet, storage temperature, geographic origin) that a laboratory-scale calibration set may not have sampled. This distinction, between direct measurement and indirect prediction, recurs throughout the review and is treated explicitly wherever a specific sensing or AI application is discussed and again at greater length in Part II in the context of food-safety risk management specifically. Table 2 summarizes these attributes alongside the sensing modality or modalities currently reported to access them and flags, attribute by attribute, whether current evidence supports direct measurement or only statistical prediction and the readiness level reported for it.

## 3. Advanced Sensing Technologies for Smart Egg Processing

### 3.1. Computer Vision

Among the sensing technologies considered in this review, computer vision—conventional RGB or grayscale imaging paired with image processing and, increasingly, deep-learning classification—is by far the most industrially mature. Automated collection and washing are already standard in many commercial table-egg facilities, and computer vision simply extends that existing automation into grading based on external defects, a task that would otherwise remain dependent on human inspectors working at line speed [7]. Machine-vision crack detection has a long history. A grey-scale image-analysis method for stationary eggs reported in 1991 achieved a 96% success rate but needed about 25 s per egg, and later work extended it to rotating eggs [4,46]; neural-network classifiers of image histograms were subsequently reported, as summarized in [47], to reach about 90% accuracy at much higher speed, and color computer vision and neural networks were applied to cracks, blood spots, and dirt stains [47] and to dirt on brown eggs [48]. These early studies defined the main image-analysis tasks (cracks, dirt, blood spots) that current deep-learning systems address; what has changed is mainly speed and the use of learned features. A recent review of shell-quality testing covers this area in more detail [49]. An image-processing-based multi-flow production line for sorting eggs has also been described [32].

The methods reported in the literature range from classical image processing to fully learned deep architectures, and the review by Ahmed and colleagues summarizes a comparison of three such approaches on one task, identifying dirty and cracked eggs. A classical feature-based method, a convolutional neural network, and a semantic segmentation model returned mean precision values (over the normal, dirty and cracked classes) of 94%, 91.25%, and 85.75%, respectively, with average processing times of 0.049, 0.11, and 0.47 ms per egg image [7,50]. Taken at face value, the classical method was both the most precise on average and the fastest. The 91.25% value is the figure stated in the conclusions of the primary study; the class precisions in its own Table 3 average 91.0% [50]. In the primary study, however, the segmentation model had the highest crack recall (0.99 against 0.88 for the classical method), and the test set comprised only 70 images [50]. The comparison illustrates a limitation that recurs in the sensing literature: accuracy figures reported across different studies, datasets, and even different methods evaluated within the same study are not automatically comparable to one another. They are shaped by the class balance, by how defect severity happens to be distributed in the test set, and by whatever error costs the developers chose to optimize against. A classical method tuned on a dataset with few borderline cases can outperform a deep model trained on a harder, more representative one, and such a comparison says little about generalization to an unseen production line.

**Table 4:** Machine-learning and deep-learning approaches for egg quality/safety prediction discussed in this review, with dataset size and validation practice made explicit.

Study	Sensing Input	Algorithm(s)	Task	Dataset Size	Train/Test or Validation Approach	Reported Performance	Highest Validation Tier Supported (Section 2)
Yao et al. [37]	Transmission Vis-NIR HSI (401–1002 nm)	IRIV wavelength selection + regression/classification	Freshness (Haugh unit), scattered yolk, eggshell cracks	Not reported	Not reported	Not reported as a single summary figure	Not determinable from reviewed source
Yao et al. [51]	Transmission Vis-NIR HSI + CARS wavelength reduction	Simplified regression models	S-ovalbumin content (freshness biomarker)	Not reported	Not reported	Not reported as a single summary figure	Not determinable from reviewed source
Li et al. [31]	HSI (1000–2500 nm)	Attention-enhanced 3D-CNN	Eggshell crack detection	400 (self-constructed)	Not reported in detail	F1 = 75.49%	Not determinable (self-constructed dataset)
Chen et al. [54]	Hyperspectral imaging with band selection	DNN; 1D/2D/3D-CNN; MobileNet	Freshness and defect inspection	1800 eggs (1000 for freshness, 800 with defects)	No train/test split or cross-validation described	99% (freshness); 100% (defects); 95% at 31 ms	Not determinable (no validation design described)
Yang et al. [25]	RGB computer vision	RTMDet (detector) + random forest	5-class grading; weight regression	Not reported (single research platform)	Not reported in detail	94.8% accuracy; R^2^ = 0.960	Not determinable (single research platform)
Liu et al. [35]	Raman spectroscopy (eggshell)	PLS-DA	Freshness classification	Not reported	Not reported in detail	Reported via confusion matrix, not a single summary figure	Not determinable
Davari et al. [42]	Micro-Raman spectroscopy (shell and yolk)	PLS-DA	Temperature/storage-time classification	Not reported	Calibration/validation split	80% (validation set)	Not determinable (validation set reported; origin not described)
Yimenu et al. [39]	Fast-GC electronic nose	Not specified beyond chemometric discrimination	Freshness discrimination, storage-time prediction	35 calibration + 28 validation	Independent calibration/validation sets	Not reported as a single summary figure	Tier 3 (independent calibration/validation sets, same study)
Li et al. [41]	Electronic nose + wavelet feature extraction	LDA, PNN (classification); SVM, BPNN (regression)	Storage-time classification; yolk-index regression	Not reported	Training/testing split reported for regression	PNN: 92.86% (test); SVM: R^2^ = 0.9641 (train)/0.8339 (test)	Tier 3 (held-out test set, same source)
Zhang et al. [55]	E-nose + HSI	MDFA-Net/adaptive fusion	Egg-quality classification	Not stated in reviewed abstract	Multimodal fusion; internal evaluation	99.88% accuracy; 99.87% precision; 99.88% recall; 99.90% F1	Not determinable (split not described in the abstract)
Hassan et al. [56]	Image + shape + weight	CNN feature extraction + ensemble ML	Grade and freshness prediction	186 brown eggs	k-fold cross-validation; public dataset/code	82.24% (SD 2.41) grade; 70.41% (SD 3.20) freshness; mean of five seeds	Tier 2 (k-fold cross-validation; public dataset; SMOTE and PCA applied before folds as described, so leakage cannot be excluded)
Ahmed et al. [57]	NIR spectroscopy	RF and other ML + SHAP	Eggshell-strength prediction	145 retail eggs (white and brown; several brands)	Random 70:30 split (100/45); fixed random state; RFE variable selection	Rp^2^ = 0.83; RMSEP = 1.49 N; RPD = 2.44	Tier 3 (random hold-out; heterogeneous retail eggs)
Ahmed et al. [58]	Vis-NIR HSI (374–1015 nm)	PLSR, RF, XGB, SVR + SHAP	Egg yolk-ratio prediction	600 white-shelled eggs; two flocks, one farm	Random 50/25/25; same flock and campaign	R^2^ 0.79, 0.73, 0.68; test RPD 1.76	Tier 3 (independent test set within study, same source)
Ong et al. [53]	HSI (450–1100 nm)	DC wavelength selection + CNN	Egg freshness prediction	381 brown-shelled eggs; one farm; stored 0–28 days	255/126 split by Haugh-unit ranking; same campaign	r = 0.9056; RMSEP = 4.4152 (Rc = 0.9173; RMSEC = 4.2305); 11 wavelengths	Tier 3 (same campaign; selection leakage not excluded)
Liu et al. [12]	NIR-HSI (1000–2500 nm)	CARS + MT-CTSE-Net	Variety; storage period	300 eggs; 3 varieties; days 1, 20, 40	Random 80/20, stratified; same campaign	Variety ~86%; period 84.2–86.4%; single-task variety 81.2% to 70.4% (day 1 to 40)	Tier 3 (same campaign)
Rouhollahi et al. [13]	UV–Vis–NIR transmittance (200–1000 nm)	PCA + MLP-ANN	S-ovalbumin; fresh/old class	300 eggs; one farm; 21 days	Random 70/10/20; classes from clustering of same eggs	S-ovalbumin R = 0.93; fresh/old 92.00%; 3 classes 79.85%	Tier 3 (same campaign)
Akbarzadeh et al. [14]	Microwave, waveguide (0.9–1.7 GHz)	PLS; ANN; SIMCA	Quality indices; storage day	244 eggs; one farm; 1–24 days	Random 80/20 (75/25 for classes)	RPD 3.50 (YC), 3.00 (ACH), 2.41 (TAH), 2.03 (HU), 1.83 (pH); storage day 100%	Tier 3 (same campaign)
Akbarzadeh et al. [15]	Microwave, free space (8–12 GHz)	PLS; CARS + ANN	ACH, YC, TAH, HU, pH	252 eggs; one farm; 1–24 days	Random 75/25; selection guided by test set	Best RPD 4.80 (ACH), 4.00 (YC), 3.27 (TAH), 3.03 (HU), 3.72 (pH)	Tier 3 (test-guided selection)
Nakaguchi et al. [16]	Thermal imaging (quail eggs)	YOLOv8x-seg; Mask R-CNN	Air-cell proportion; freshness grade	1600 quail eggs; 600 for calibration and test	Thresholds derived and tested on same 600 eggs	Sorting accuracy 67.5% and 75.8%; F1 0.719 and 0.778	Tier 1 (same eggs)
Al-Mamun et al. [17]	Transmission imaging; Vis–NIR (400–1000 nm)	Threshold; LDA, QDA, SVM	Blood spots; runny yolk (induced)	800 eggs; white and brown; 100 per class	Stratified 5-fold cross-validation	Blood spots 97.50% and 98.37%; runny yolk 99.01% and 100.00%	Tier 2 (induced defects only)
Akowuah et al. [18]	Handheld NIR (740–1070 nm)	MSC-PCA; LDA; PLS-R	Freshness category; date of lay	120 eggs; one farm; 630 spectra; two storage conditions	472/158 calibration/prediction split of spectra; independent set also reported	LDA > 95%; date of lay R 0.87–0.88; RMSEI 2.57–2.66 days	Tier 3 at most (three scans per egg)
Han et al. [19]	Vis-NIR reflectance	PCA-BP; SIMCA	Color, crack and feeding mode	720 eggs; one locality; cracks induced by bumping	2/3 validation, 1/3 test	Crack/intact 100% (pink, green), 98.75% (white)	Tier 3 (same campaign; induced cracks)
Shi et al. [20]	Electrical current signal	RF, SVM, LDA, DT, K-means	Microcrack detection	770 eggs (367 intact, 403 artificially cracked); 267 duck eggs	Test split not described	RF 99.44%; duck eggs 98.16%	Not determinable (split not described)
Pakuła et al. [21]	Fiber-optic multispectral reflectometer	Neural network (SOTA)	Origin: healthy vs Mycoplasma-infected flock	2521 shell measurements; healthy from one reference flock, infected from commercial flocks	Random 7:3 split of measurements, repeated 100 times	F-score about 99% (white and brown)	Tier 2 (flock and repeated measurements confounded)
López Vargas et al. [22]	X-ray computed tomography (3D)	U-Net 3D; FCN 3D	Egg morphometry (height, width, shell thickness, volume)	150 eggs, 0–28 days; 122 CT volumes	80/10/10 split of volumes	Accuracy up to 98.69%	Tier 3 (same campaign; not a freshness sensor)
Nukala et al. [52]	HSI (400–1000 nm)	RF and other regressors; nested CV	Freshness grading and HU prediction	1500 eggs from 3 breeds; 3 temperatures	Leakage-free preprocessing; 5×3 nested CV	>92% at 3 °C; >88% at 20 °C; >85% at ~33 °C; R^2^ up to ~0.95	Tier 2 (nested cross-validation; 3 breeds, 3 temperatures)
Bischof et al. [28]	1H NMR of yolk extract (destructive sample preparation)	LDA, QDA, PLS-DA, SVM, RF, kNN, ANN	Housing-system authentication	4188 development eggs; 290 retail eggs (external set)	80/20 split; 10-fold CV; retail eggs as external set	CV 84.7–98.5%; test 84.6–99.0%; external 45.9–62.8%	Tier 4 (independent-source; retail labels unverified; three classes)

The more recent trajectory in the field has been toward deep convolutional architectures capable of handling external defect detection and grading jointly, rather than as separate steps. Yang and colleagues, for instance, developed a two-stage system pairing a real-time multitask detector (RTMDet) for classification with a random forest model for weight prediction, evaluated on cage-free egg production and able to sort eggs into five categories: intact, cracked, bloody, floor, and non-standard size. The system reached a best classification accuracy of 94.8% and an R^2^ of 0.960 for weight prediction, and the authors position it as among the first to perform classification and weighing within a single pipeline [25]. It should be noted that this evidence comes from a university research platform at a laboratory and pilot scale, not from validation across commercial facilities, and the authors themselves frame the work as a proof of concept rather than a deployment-ready system. Even so, it points to where the field is heading: toward multitask models that consolidate several single-purpose classical vision steps into one learned pipeline.

Acoustic-impulse analysis is the best-established non-optical approach to crack detection. In this method, the frequency response of a lightly tapped egg is analyzed, because an intact shell responds with a repeatable frequency pattern and a cracked shell does not [59].

Commercial deployment is concentrated in the external-grading step. Manufacturer descriptions of current high-capacity graders list automated weighing, dirt detection, and crack detection, with throughput of up to 180,000 eggs per hour for one 18-row machine [60] and contactless vision-based crack detection offered as an integrated module on another [61]. Acoustic-impulse crack detection was developed for high-speed lines, where manual candling above about 10,000 eggs per hour per lane had become a bottleneck; the original study reported 90% crack detection with fewer than 0.5% false rejects [59], and later work combining acoustic features with supervised classifiers reported 97.1% identification in a prediction set [62]. These sources describe equipment capability. They are manufacturer or laboratory reports, and independent peer-reviewed accuracy audits of commercial crack detectors were not identified for this review. Internal quality, composition, and microbial safety are still assessed on commercial lines by sampling and destructive tests, and the spectroscopic, e-nose, and biosensor studies reviewed below remain at a laboratory or pilot scale.

Computer vision is limited by its nature to surface information. It can characterize the shell, and, through candling-style transillumination, resolve coarse internal features such as air cell size or gross yolk displacement, but it cannot access the chemical composition, cannot resolve finer internal quality indices such as the Haugh unit without destructive calibration, and cannot detect microbial contamination directly. This gap motivates the spectral and hyperspectral methods described next.

### 3.2. Hyperspectral Imaging

Hyperspectral imaging (HSI) combines the spatial resolution of a camera with the chemical specificity of spectroscopy, acquiring a reflectance or transmittance spectrum at every pixel across a wide contiguous wavelength range, and it is regarded in recent reviews as a leading candidate for non-destructive internal egg-quality assessment [7,11]. Because HSI captures both spatial and spectral information simultaneously, it can in principle localize a defect (where on the shell a crack sits) while also characterizing the composition (the chemical basis of a freshness change), a combination neither pure imaging nor pure point-spectroscopy offers on its own.

Yao and colleagues’ 2022 study illustrates how one hyperspectral dataset can serve several tasks. Working with transmission visible-near-infrared hyperspectral images collected across 401 to 1002 nm, they used standard normal variate preprocessing and an iteratively retained informative variable algorithm for wavelength selection, then applied the resulting models to three distinct tasks from one dataset: predicting egg freshness (via Haugh unit), detecting scattered (broken) yolk, and detecting eggshell cracks, the last through a purpose-built algorithm for extracting the geometric features of crack patterns in the images [37].

A companion study, conducted by a partially overlapping author group, applied the same transmission HSI approach to a more targeted question: tracking S-ovalbumin content, a recognized freshness biomarker, over the course of egg storage [51]; the simplified models that it produced are discussed in Section 3.3. Both studies, however, share the same evidentiary limits: they represent laboratory-scale, single-site validation, and neither the underlying dataset sizes nor the extent of external validation across eggs from different flocks, feeds, or storage histories are reported in the material examined here. This is a substantive gap, because egg composition is known to depend on the hen strain, age and diet; without cross-population validation, it is unclear how far these models generalize beyond the conditions under which they were built. More recent HSI studies extended the approach across breeds and storage temperatures [52] and combined it with deep learning and distance-correlation wavelength selection [53]; both are examined in Section 5.1. Earlier near-infrared HSI work in the 900–1700 nm range predicted the Haugh unit of eggs stored at 25 °C [63]. Acquisition geometry also affects performance. In a study of hyperspectral scattering images combined with ensemble learning, the highest classification accuracies were 100% for scattering, 96.25% for mixed, 95.0% for transmission, and 88.75% for reflection images [64]. These values come from one study with one instrument and one sample set, so the ranking of geometries cannot be assumed to hold elsewhere.

Crack detection is one instance where HSI’s added spectral dimension yields a demonstrable, if still partial, advantage over conventional imaging. A recent study paired hyperspectral data spanning 1000–2500 nm with an attention-enhanced three-dimensional convolutional neural network, termed 3D-CrackNet, specifically to identify eggshell cracks that are difficult to detect under conventional lighting, particularly on brown or multi-colored shells and against visually complex backgrounds. The reported F1-score was 75.49%, on a self-constructed dataset of 400 egg samples [31]. The authors frame their motivation directly in relation to the Yao et al. approach discussed above, observing that conventional HSI-based crack detection still struggles with fine crack tips, whose faint contrast against the surrounding shell makes precise localization difficult [31]. That an F1-score in the mid-70s is presented as an advance suggests that automated detection of fine eggshell cracks remains unresolved, even with the added spectral information, whereas external defect classification is more mature. The 400-sample dataset was self-constructed, and the reported performance has not been tested on an independent egg population.

Across the HSI literature applied to eggs, three limitations recur with some consistency. The first is geometric: the curved, glossy shell surface scatters and specularly reflects light in ways that complicate both image segmentation and spectral interpretation, and most studies address this through carefully controlled illumination geometries, a solution that may not translate cleanly to the less controllable conditions of a high-speed production line. The second concerns pigmentation, which acts as a confound rather than a purely cosmetic variable: brown and white shells absorb and transmit light differently across the visible and near-infrared range, so that a model calibrated on one shell color typically requires separate calibration, or at minimum careful independent validation, before it can be applied reliably to the other. The third and probably the most consequential for industrial translation is economic and computational: hyperspectral systems are more expensive than conventional RGB cameras, and the data cubes that they generate are large and pose a throughput problem for real-time processing at commercial line speeds. A 2025 review focused specifically on hyperspectral imaging in the egg and hatchery industry examines this readiness gap in some depth [11]. That review does not address the link to downstream process selection, which is outside its scope.

### 3.3. Multispectral Imaging

Multispectral imaging (MSI) is a possible route around the throughput limitation of hyperspectral imaging: rather than acquiring a continuous spectrum at every pixel, an MSI system captures reflectance at a small number of pre-selected, discrete wavebands, trading spectral resolution for acquisition speed and cost. What is notable in the egg literature specifically is that dedicated, purpose-built multispectral hardware is comparatively rare; instead, most of what functions as multispectral sensing for eggs has emerged as a second stage of hyperspectral research, in which chemometric wavelength-selection algorithms are used to identify which handful of bands out of several hundred actually carry the discriminating information, so that a much simpler and faster instrument could in principle be built around just those bands. Yao and colleagues’ S-ovalbumin monitoring study is explicit about this logic, using competitive adaptive reweighted sampling to reduce hundreds of hyperspectral wavelengths down to a small feature set, with the stated aim of developing simplified models suited to an online multispectral system rather than a full hyperspectral one [51].

Band selection has also been combined with deep networks for real-time inspection. Chen and colleagues developed hyperspectral egg defect and freshness inspection techniques that combine band selection with deep neural networks, one-, two- and three-dimensional convolutional networks, and MobileNet. They reported overall accuracies of 99% for freshness and 100% for defect inspection and, in a faster defect-inspection configuration with an inspection time of 31 ms, an overall accuracy of 95%; the work was framed around both production-line and consumer use [54]. The abstract does not state how test data were separated from training data, so no validation tier is assigned (Table 4). A broader review of spectral imaging applied to poultry products more generally (not eggs specifically) reached a comparable conclusion about the field as a whole: hyperspectral and related spectral-imaging methods are well represented in the literature across fecal-matter detection, food fraud, microbiological contamination, physical defects, and product quality, but the review’s own synthesis, built from a systematic search of 67 studies, still treats these largely as laboratory demonstrations rather than deployed systems [65]. The practical conclusion for this review is that multispectral imaging for eggs is best understood not as a rival technology to hyperspectral imaging but as its industrially oriented endpoint, the wavelength-reduced, cost-reduced version a hyperspectral calibration is meant to produce once the informative bands have been identified.

### 3.4. Near-Infrared Spectroscopy

Near-infrared (NIR) spectroscopy, typically applied as point spectroscopy through the shell rather than as an imaging modality, has been applied to egg freshness for well over a decade [33,34]. Giunchi and colleagues demonstrated the non-destructive freshness assessment of shell eggs using Fourier-transform NIR spectroscopy with a fiber-optic probe, establishing that the technique could track storage-related deterioration through an intact shell [33]. Eggs were stored for 16 days at 20 °C; test-set R^2^ values were 0.722 for air cell height, 0.789 for the thick albumen height, and 0.676 for the Haugh unit, and eggs were classified by storage day with 100% accuracy [33]. All eggs were stored at a single temperature. Building on this, Abdel-Nour and colleagues used visible/NIR spectroscopy specifically to predict the Haugh unit, air cell height, and other albumen-quality indices, positioning NIR as a candidate replacement for the destructive reference measurements that it aims to supersede [34]. A consistent finding runs through this body of work, echoed in more recent studies that use chemometric wavelength- and variable-selection algorithms [66,67]: NIR spectroscopy can track the same underlying biochemical trajectory, albumen thinning, pH rise, and moisture migration that defines the classical Haugh unit and yolk index, without requiring the egg to be broken.

NIR’s principal advantages are instrumental rather than purely analytical: portable, handheld, and fiber-optic-probe NIR systems are commercially available, cheaper than hyperspectral cameras, and require no imaging optics, so point NIR is one of the more plausible options discussed here for retrofit onto an existing candling or grading line. Portable NIR instruments have been tested on intact eggs: Cozzolino et al. analyzed 700 eggs from two production systems (cage and free-range) stored at room or cold temperature and predicted storage time with a cross-validated R^2^ of 0.67 to 0.84 and standard errors of cross-validation of 5.4 to 7.6 days; the weakest result (R^2^ 0.67, RPD 1.8) was for cage eggs at room temperature [68]. Portable NIR with machine learning has also been applied to quail eggs [69]. Transmittance spectroscopy in the ultraviolet–visible range (200–1000 nm) predicted the S-ovalbumin content in 300 chicken eggs stored for 21 days at two temperatures, with a test-set correlation of 0.93, and separated fresh from old eggs with 92.00% overall accuracy on a random 70/10/20 split of one campaign (Tier 3); the fresh/old classes were derived through fuzzy clustering of the same eggs’ destructive indices, and overall accuracy fell to 79.85% with three classes [13]. A handheld NIR spectrometer (740–1070 nm) identified the freshness category of eggs stored under ambient or cold conditions with identification rates above 95% and predicted the marked date of lay with R = 0.87–0.88 (RMSEI 2.57–2.66 days) based on an independent set, but it used 120 eggs from one farm, each scanned three times, so several spectra come from the same egg and Tier 3 is the most this design supports [18]. Its principal limitation is the mirror image of that advantage: because a conventional NIR spectrometer collects a single averaged spectrum rather than a spatially resolved image, it cannot localize where on the shell or where within the egg a defect or compositional gradient occurs, a task at which hyperspectral and computer-vision systems (Section 3.1, Section 3.2 and Section 3.3) are inherently better suited. This complementarity, spatial resolution from imaging modalities, chemical information, and low cost from point spectroscopy, is one of the clearest arguments in the sensing literature for multimodal rather than single-technology sensing architectures, a theme developed in Section 3.10.

Two studies illustrate how the measurement geometry and storage conditions limit NIR performance. Wang et al. compared diffuse reflection with diffuse transmission on 210 brown-shell eggs stored at 4 or 25 °C. Transmission was the more informative mode, because in reflection mode, the signal came mainly from the shell membrane. Linear discriminant analysis identified the storage day with 91.4% accuracy in the prediction set at 25 °C but only 25.6% for refrigerated eggs, and the synergy interval PLS predicted the Haugh unit with an RMSEP of 4.25 at 25 °C [67]. Lin et al. found that successive projections and interval-based variable selection reduced the number of wavelengths needed to model egg freshness; the best model combined a genetic algorithm with the synergy interval PLS, used 42 variables, and reached an RMSEP of 3.29 [66]. Both studies were laboratory studies on small sample sets, and the tier of their validation is not stated in the abstracts reviewed here. The NIR studies differ in the measurement geometry (reflection, transmission, fiber-probe contact), storage temperature, sample size, and target (Haugh unit, storage day, storage time), so their performance figures cannot be compared directly. In the abstracts reviewed here, none describes validation on eggs from a different facility or season, and the calibration models are specific to the instrument and storage conditions used; transfer to other instruments, flocks, and storage histories would require recalibration or calibration transfer, which these studies do not examine. Mid-infrared (MIR) spectroscopy is considered separately because its sampling requirements differ. MIR bands are fundamental vibrations and are more specific than the NIR overtone and combination bands, but penetration is very short and water absorbs strongly, so egg studies of MIR use attenuated total reflection (ATR-FTIR) on sampled material. Michalczyk and Kurczab recorded the ATR-FTIR spectra of shell fragments, albumen, and yolk from 50 eggs (10 per laying-system class, one egg per class on each of 10 days over 29 days of storage) and used cluster analysis and PCA to separate classes and follow compositional change [70]. Albumen and yolk were sampled from broken eggs, so that work is not an intact-egg measurement, the sample is small, and no predictive model with independent validation is described. No study of MIR on intact eggs at line speed was identified, and based on current evidence, MIR is not a candidate for in-line deployment.

### 3.5. Raman Spectroscopy

Raman spectroscopy probes molecular vibrational modes and, because its scattering signal is largely insensitive to the water content, offers a chemically specific window onto the egg composition that complements the water-dominated absorption spectra that NIR spectroscopy relies on. Applied directly to the eggshell surface, Raman spectroscopy has been shown to track the same cuticle-degradation process that governs freshness loss: Liu and colleagues demonstrated an online, non-destructive egg-freshness assessment from the shell using Raman spectroscopy combined with chemometrics, reporting that predictive performance was strongest when spectra were collected from the shell regions in direct contact with the albumen, consistent with a cuticle-protein-degradation mechanism, and building a partial least squares discriminant analysis classification model whose confusion-matrix performance the authors related directly back to that mechanistic interpretation rather than treating the model as a black box [35]. Independent work applying micro-Raman spectroscopy to both shell and yolk reported an 80% validation-set classification accuracy for temperature- and storage-time-based egg conditions, with carotenoid-associated Raman peaks identified as the dominant discriminating feature and relating the classification to chemical changes (carotenoid depletion and lipid and protein oxidation) [42].

Raman models can be interpreted through specific peaks, such as carotenoid or protein amide bands, which is an advantage over some deep-learning imaging approaches whose driving features are less directly interpretable. Raman spectroscopy has also been proposed for egg authentication, distinguishing eggs from different rearing systems (cage, barn, free-range, and organic) on the basis of carotenoid, lipid, and protein Raman-intensity ratios [71], an application category directly relevant to the adulteration and authenticity attributes introduced in Section 2.4. Against these strengths, Raman signals from biological samples are frequently confounded by the fluorescence background, with egg pigments and porphyrins being a particularly relevant source of that interference for eggs, and, as with the other spectroscopic modalities reviewed here, the calibration models reported to date have been developed and validated on single, laboratory-controlled sample sets rather than across multiple flocks, seasons, or facilities. The Raman studies discussed here report calibration or validation results from one sample set each (Table 4), and none tests transfer to other flocks or facilities.

### 3.6. Fluorescence Spectroscopy

Fluorescence spectroscopy exploits the fact that several of the egg’s intrinsic biochemical constituents, tryptophan and other aromatic amino acids, vitamin A, and porphyrin derivatives associated with the eggshell cuticle, are natively fluorescent and that the intensity and spectral position of their emission shift measurably as an egg ages. Karoui and colleagues’ two-part study on front-face fluorescence spectroscopy is the most detailed treatment of this approach identified here: working first with thick and thin albumen, they found that classification based on tryptophan fluorescence achieved only modest accuracy (57.1% and 51.9% correct classification in calibration and validation, respectively) [36], and then, working with egg yolk, reported 94.9% and 91.4% correct classification in calibration and validation using vitamin A fluorescence [40]. The gap between these results suggests that, within one modality, the choice of target fluorophore can matter as much as the choice of technology; however, the two studies used different egg fractions (albumen and yolk), so the comparison is indicative only. Separately, cuticle-associated porphyrin fluorescence, detectable at the shell surface, has been proposed as a freshness-correlated signal accessible without probing the egg’s interior at all [8,72]. The institute report cited there found that the red autofluorescence of the shell, attributed to porphyrin, was stronger in fresh eggs than in older ones [72]. Both Karoui studies report calibration and validation results from one study population, and no independent external test is described in the material examined here.

Fluorescence spectroscopy shares the core non-destructive advantage of NIR and Raman spectroscopy and adds one further benefit: because fluorescence measurements can be highly sensitive at low analyte concentrations, detection limits can in principle fall below those of absorption-based techniques targeting the same compound. Its main practical constraint, suggested by the gap between the tryptophan and vitamin A results above (which came from different egg fractions), is that the fluorescence signal strength and specificity vary substantially with the target fluorophore, storage condition, and excitation wavelength. A fluorescence-based sensing system therefore demands more careful, compound-specific optimization than a broadband absorption or scattering technique might require.

### 3.7. Electronic Nose and Volatile Sensing

Electronic nose (e-nose) systems use arrays of gas sensors, or in some configurations, fast gas chromatography, to generate a composite “smell fingerprint” from the volatile organic compounds that an egg releases through its shell pores as it ages or spoils. Among the sensing categories considered here, this is the one most directly targeted at the chemistry of spoilage itself, rather than at physical or optical proxies for it. Dutta and colleagues’ early work established the basic feasibility of this approach for egg freshness determination using a conducting-polymer sensor array [38]. Building on that foundation, Yimenu and colleagues used a fast gas-chromatography e-nose to discriminate freshness and predict the storage time, sensory quality, and internal quality changes over a 20-day storage period, evaluating their models on independent calibration (35 samples) and validation (28 samples) sets. An explicit train/validation split of this kind is not universal in the egg-sensing literature (Section 4.4) [39]. Li and colleagues took a complementary, more quantitative approach, combining e-nose signals with wavelet-transform feature extraction to predict both the storage time and yolk index. Their classification models, linear discriminant analysis and a probabilistic neural network, reached up to 92.86% correct classification of storage-time categories on a held-out test set, while, among their regression models, a support vector machine outperformed a back-propagation neural network for yolk-index prediction (R^2^ of 0.9641 in training versus 0.8339 in testing) [41]. The drop from training to testing R^2^ illustrates the generalization gap discussed in Section 4.4.

Volatile sensing’s clearest conceptual advantage is that it targets a signal, the accumulation of amines, aldehydes, and other volatile metabolites, mechanistically closer to actual spoilage than most optical proxies are. Its clearest limitation lies in throughput and specificity. Gas-sensor arrays typically require a headspace equilibration period of minutes rather than the sub-second acquisition times reported for imaging-based methods, complicating integration into a high-speed grading line. Sensor drift and cross-sensitivity to ambient humidity and temperature remain recognized practical obstacles to field deployment as well, though the egg-specific literature considered here treats these as general operational concerns rather than quantifying their impact directly.

### 3.8. Biosensors and Electrochemical Sensors

Biosensors, devices that couple a biological or biomimetic recognition element (an antibody, aptamer, bacteriophage, or DNA probe) to an electrochemical, optical, or piezoelectric transducer, occupy a different niche from the spectroscopic and imaging technologies discussed above. Rather than inferring safety risk from an indirect physical or chemical proxy, they are designed to bind a target pathogen or pathogen-associated molecule directly, placing them closer to the “measurement” end of the measurement-versus-prediction distinction introduced earlier. A recent comprehensive review of electrochemical Salmonella-detection methods surveys these methods and includes several biosensors evaluated specifically on egg or eggshell matrices. Among them is a wireless magnetoelastic biosensor using bacteriophage-based recognition, reported to detect Salmonella Typhimurium on eggshell samples with a limit of detection of 1.6 × 10^2^ CFU per square centimeter under controlled humidity conditions, alongside immunosensor-based approaches validated against egg and poultry-meat samples spiked with known Salmonella concentrations [43]. Surface plasmon resonance biosensors have also been used to detect egg-yolk antibodies indicative of prior Salmonella Enteritidis infection in the laying hen [73], a flock-surveillance application distinct from direct pathogen detection in the egg product itself, though it is worth noting explicitly that a positive antibody signal indicates prior host exposure, not current product contamination, and should not be conflated with the direct pathogen-detection biosensors discussed above.

The evidentiary picture for egg-specific biosensors is, at present, laboratory proof of concept. The studies synthesized in the review cited above validate their sensors against spiked samples with deliberately introduced, known bacterial concentrations, a necessary first step but one that does not establish performance against the lower, more variable, and matrix-confounded contamination levels that would be encountered on an actual shell-egg or liquid-egg processing line. None of the egg-relevant biosensor studies identified for this review report validation across multiple production facilities, multiple *Salmonella* serovars in combination, or the complex protein- and lipid-rich matrix of whole liquid egg, all considerations that would need to be addressed before a biosensor could be described, in the terms of the evidence hierarchy adopted later in this review, as more than a laboratory proof-of-concept.

Three categories should be kept apart in this section and in later parts of the review. Direct pathogen detection means a sensor or assay that targets the organism or a pathogen-specific marker. Indirect contamination-risk prediction infers risk from proxies such as cracks, the cuticle condition, or volatile profiles; it cannot confirm that Salmonella is present or absent. Laboratory biosensor assays detect pathogens in swabs, rinses, enriched cultures, or homogenates, so they need sample collection or preparation and are not non-destructive assessments of an intact egg. A high accuracy for a proxy-based model is not evidence that eggs are free of pathogens, and such a model should not be used as a release criterion for food safety.

### 3.9. Other Emerging Sensing Technologies

A smaller set of additional modalities appears in the egg-sensing literature with enough frequency to warrant brief mention, though the evidence base for each remains thinner than for the technologies discussed above. Infrared thermal imaging has been explored as a low-cost, camera-based approach to freshness classification [74]; in quail eggs, thermal imaging of the air cell with YOLOv8x-seg and Mask R-CNN reached sorting accuracies of 67.5% and 75.8% on 600 eggs whose air-cell thresholds had been calibrated on the same eggs (Tier 1) [16]; dielectric properties combined with image features have been used to predict egg quality [75]; microwave spectroscopy, in a waveguide and in a free-space arrangement, predicted the air cell height, yolk coefficient, thick albumen height, Haugh unit, and albumen pH in single-farm studies of 244 and 252 eggs with random calibration/test splits (Tier 3), with a best residual predictive deviation of 3.50 in the waveguide study and 4.80 in the free-space study, whose choice of spectrum, orientation, and model was guided by test-set performance as described [14,15]; radiographic (X-ray) imaging has been tested for table-egg grading [76]; computed tomography with 3D deep-learning segmentation estimated the egg height, width, shell thickness, and volume in 150 eggs stored for up to 28 days, with segmentation accuracy up to 98.69% on an 80/10/10 split of 122 image volumes (Tier 3), which is morphometry rather than a freshness sensor [22]; and low-field nuclear magnetic resonance (LF-NMR) has been investigated for changes in bound and free water as albumen thins with age [8,77]. Terahertz spectroscopy and imaging and ultrasound-based assessment appear more sporadically in the literature, generally surfacing in single studies rather than forming an established sub-literature of their own; examples are terahertz measurement of eggshell thickness [78] and ultrasound phase velocity as a freshness indicator [79]. Acoustic-impulse methods are the exception for eggshell cracks, where a sub-literature dating to around 2000 exists [59,62], although it concerns shell integrity rather than internal quality. Several recent reviews cover these modalities in more detail than is possible here [8,10,77], and a consistent thread runs through them: none of these emerging modalities has yet accumulated the depth of independent replication seen for hyperspectral imaging, NIR spectroscopy, or computer vision. At this stage, they are best regarded as candidate additions to a multimodal sensing architecture rather than as independently validated technologies in their own right.

### 3.10. Cross-Technology Synthesis

Three patterns emerge from Section 3.1, Section 3.2, Section 3.3, Section 3.4, Section 3.5, Section 3.6, Section 3.7, Section 3.8 and Section 3.9. First, there remains a trade-off between spatial and chemical information: imaging methods localize defects and compositional gradients, whereas point spectroscopies and volatile sensing provide chemically informative signals without the same spatial detail. This complementarity provides a strong rationale for multimodal architectures. Multimodal fusion has also been demonstrated in eggs. Zhang et al. combined electronic-nose and hyperspectral information for egg-quality classification, while Hassan et al. more recently fused image, shape, and weight features for non-destructive grading and freshness prediction [55,56]. A 2026 Foods review has also synthesized the growing integration of AI with VIS-NIR, Raman, fluorescence, and HSI and explicitly identifies multi-sensor fusion as a future direction [80]. The evidence therefore supports the conclusion that multimodal fusion has been demonstrated but remains fragmented, task-specific, and insufficiently validated across independent production environments. Second, the measurement-versus-prediction distinction remains fundamental: computer vision and pathogen-recognition biosensors can approach direct measurement, whereas most spectroscopic and e-nose systems infer a target attribute from a correlated physical or chemical signal. Third, the strongest unresolved issue is generalization. Even when multimodal or sophisticated deep-learning models perform well internally, the literature still provides limited evidence across independent facilities, seasons, breeds, geographies, sensor configurations, and production-line conditions. The field’s central challenge has consequently shifted from proving that multiple signals can be fused to proving that the resulting models remain reliable when the data-generating environment changes. Table 3 consolidates the technology-by-technology evaluation developed across Section 3.1, Section 3.2, Section 3.3, Section 3.4, Section 3.5, Section 3.6, Section 3.7, Section 3.8 and Section 3.9 into one comparative summary.

## 4. Artificial Intelligence and Machine Learning in Egg Processing

Section 3 already introduced, in passing, most of the specific algorithms that this section now examines directly: partial least squares regression and its discriminant variant, support vector machines, random forests, extreme gradient boosting, and a handful of convolutional architectures all appeared attached to particular sensing studies. That section was organized by sensing technology. This section asks how a sensor output, however acquired, should be converted into a quality or safety prediction, and on what basis that conversion can be trusted.

### 4.1. Classical Machine Learning

The great majority of egg-quality and egg-safety models published to date are built on what this review, following common usage, terms classical machine learning: partial least squares regression and its discriminant classification counterpart (PLS-DA), principal component analysis used either for dimensionality reduction, or, combined with a discriminant step, for classification, support vector machines (SVM) and support vector regression (SVR), random forests, decision trees, k-nearest neighbors, and increasingly, gradient-boosted ensemble methods such as XGBoost. The appeal of this family of methods for spectroscopic and chemometric egg data is well matched to the data’s own structure: hyperspectral and NIR spectra are high-dimensional but highly collinear (adjacent wavelengths carry redundant information), which is exactly the regime PLS-based methods were designed for, and the moderate sample sizes typical of egg studies, usually in the range of a few hundred to at most a few thousand eggs, are within the range on which these methods can be calibrated reliably.

Several examples from Section 3 illustrate both the strengths and the boundaries of this approach. Li and colleagues’ e-nose study compared four classical methods head-to-head on the same dataset, linear discriminant analysis, a probabilistic neural network, a back-propagation neural network, and a support vector machine, and found that the best-performing algorithm depended on the specific outcome being predicted. A probabilistic neural network proved preferable for storage-time classification, while a support vector machine outperformed back-propagation for the continuous yolk-index regression task [41]. This kind of within-study, multi-algorithm comparison is more informative than a single reported accuracy figure, because it shows that “which classical method is best” does not have a fixed answer independent of the prediction task, the outcome type (classification versus regression), and the underlying data structure. Wavelength- and feature-selection algorithms, among them competitive adaptive reweighted sampling, the iteratively retained informative variable method, and successive projections algorithms, function in the spectroscopic literature as companions to these classifiers. They are not only a data-reduction step; the wavelengths that a selection algorithm retains can in principle be mapped onto known chemical absorption or scattering features, a property discussed in Section 4.3.

Classical methods’ comparative advantages are threefold: they generally require substantially less training data than deep architectures to reach stable performance, their outputs (a regression coefficient, a support vector, a selected wavelength) are more directly interpretable without additional explainability tooling, and they are computationally light enough to run on modest hardware. Their principal limitation is that they generally depend on hand-engineered or algorithmically pre-selected features, a chosen wavelength set, a texture descriptor, and a defined region of interest, rather than learning a representation directly from raw pixel or spectral data. This caps their performance on tasks such as detecting a faint, irregularly shaped eggshell crack against a variably pigmented background, where the discriminating pattern is not well captured by any predefined feature.

### 4.2. Deep Learning

Deep learning and convolutional neural networks (CNNs) specifically enter the egg literature primarily through computer vision and hyperspectral image classification, where the value of automatically learned spatial or spectral feature hierarchies is clearest. Yang and colleagues developed an RTMDet-based grading system that combines a real-time detector with a random-forest regression component [25], while Li and colleagues developed an attention-enhanced 3D-CNN for hyperspectral crack detection [31]. More recent work also shows that multimodal feature fusion can be implemented with relatively lightweight external attributes: Hassan et al. combined image-derived deep features with egg shape and weight, reporting mean accuracies of 82.24% for grade classification (high versus low Haugh-unit class) and 70.41% for freshness classification (fresh versus old yolk-index class) over five random seeds, on a public 186-egg dataset [56]. These results illustrate both the promise and the present scale of the evidence: multimodal deep-learning pipelines are feasible, but their datasets remain small relative to the variability encountered in commercial egg supply chains.

Transfer learning, initializing a CNN with weights learned on a large, unrelated image corpus (most commonly ImageNet) and then fine-tuning on a much smaller, task-specific egg dataset, is used in several of the deep-learning egg studies discussed here [56,81]. It is the usual mitigation for small labeled datasets, which in the studies reviewed here contain hundreds to a few thousand samples, far fewer than the tens or hundreds of thousands typically used to train a CNN from scratch. Object detection and segmentation architectures, of which RTMDet [25] is the example discussed in Section 3.1, extend this further, allowing a single model to localize, classify, and in some cases measure an egg or a defect within one inference pass, which is the architectural direction most consistent with the real-time processing-line requirements discussed in Part II.

Deep learning’s comparative advantage over the classical methods of Section 4.1 is its ability to learn discriminating features directly from raw image or spectral data, which matters most for tasks, like fine eggshell crack detection, where no hand-engineered feature reliably captures the pattern. That advantage is not unconditional: it is bought at the cost of requiring more training data, more computational resources for both training and (depending on architecture) inference, and, as the next subsection discusses, a substantially harder interpretability problem than a PLS or SVM model presents.

### 4.3. Explainable AI

Explainability has moved from a peripheral concern to an explicit design requirement in food-quality machine learning over the past few years, and a recent, food-specific review of explainable AI (XAI) techniques frames the reason clearly: increasingly complex models are needed to meet food-quality accuracy demands, but that complexity itself raises reliability concerns, and XAI remains, in the review’s own assessment, comparatively underused in food engineering relative to other application domains, which the same review argues limits how much trust model users can reasonably place in AI-based quality decisions [82]. The distinction that the same source draws between interpretability, an intrinsic property of a model whose internal logic can be inspected directly, and explainability, a post-hoc approximation generated after a (typically non-interpretable) model has already been trained, is a useful one to carry into the egg-processing context specifically [82]. Classical methods of the kind reviewed in Section 4.1, PLS regression coefficients, selected wavelengths, and support-vector decision boundaries in a low-dimensional feature space, are largely interpretable in this intrinsic sense: the wavelengths that a PLS-DA freshness model weights most heavily can be checked directly against known absorption features of water, protein, or carotenoids, which is one reason wavelength-selection algorithms function as a form of built-in explainability, as noted in Section 4.1. Deep architectures, by contrast, generally require a separate post-hoc explanation step, commonly SHAP (Shapley Additive Explanations) for tabular or spectral inputs or Grad-CAM (Gradient-weighted Class Activation Mapping) for image inputs, to identify which input regions or spectral bands drove a given prediction [82].

**Figure 2:**
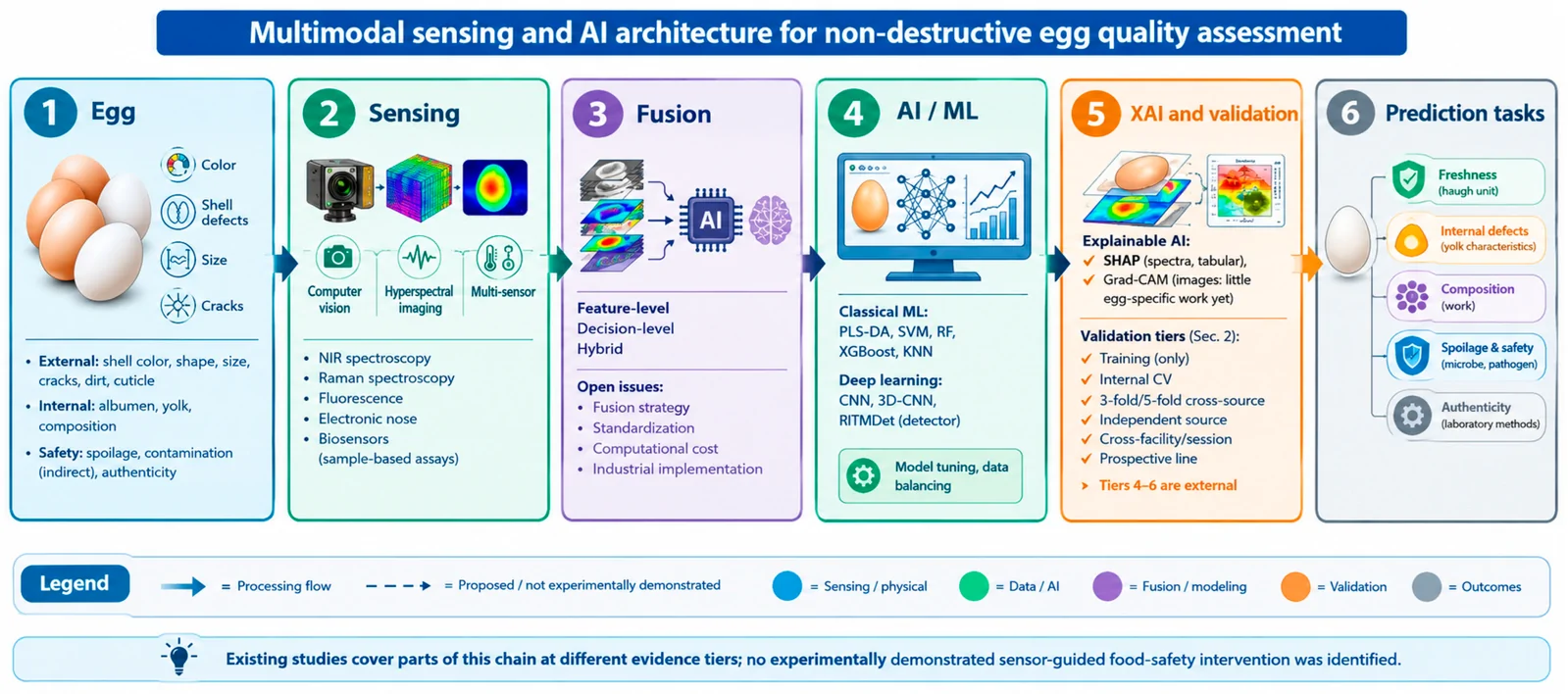
Multimodal sensing and AI-enabled architecture for non-destructive egg quality assessment. Complementary information from imaging, hyperspectral/multispectral sensing, spectroscopy, electronic nose, and physical measurements can be integrated at the feature-, decision-, or model level to improve prediction of egg quality and safety attributes. Explainable AI methods can subsequently provide interpretable links between sensor-derived features and model predictions, while independent validation is required to assess robustness and generalization across eggs, storage conditions, breeds, instruments, and facilities.

Egg-specific explainable AI is now demonstrated rather than absent. Ahmed et al. combined NIR spectroscopy with machine learning and SHAP to predict eggshell strength, with the selected variables contributing to an interpretable model of shell strength [57]. A second study combined visible-NIR hyperspectral imaging, machine learning, and SHAP for non-destructive prediction of the egg yolk ratio, with the explanation maps linking influential wavelength regions to water, lipid, and protein-related spectral features [58]. These studies materially change the framing of the explainability gap. The issue is no longer whether XAI can be applied to eggs, but how broadly, rigorously, and operationally it is being used. Current evidence remains concentrated in a small number of regression and spectroscopic applications, and the literature still provides limited evidence that explanations are validated against expert knowledge, remain stable under domain shift, or are used prospectively to detect spurious cues before deployment. For computer-vision systems in particular, egg-specific Grad-CAM-style analysis remains much less developed than the SHAP-based interpretation of tabular or spectral models. Thus, explainability should be treated as an emerging capability that requires validation, not as a solved prerequisite for trustworthy deployment. The relationships among complementary sensing modalities, multimodal data fusion, AI/ML prediction, explainability, and external validation are summarized conceptually in Figure 2.

### 4.4. Model Validation

How an accuracy figure was estimated matters more than the figure itself, because the estimation procedure determines whether the result applies outside the dataset it came from. A recent review spanning machine and deep learning applications in food integrity found overfitting to be widespread, particularly among deep architectures trained on small datasets, in many cases under 100 samples. A second, compounding problem emerged alongside it: external validation on independent cohorts is often skipped entirely. The consequence, as that review argues, is a body of literature reporting accuracy figures that look strong on paper but do not hold up in real-world implementation [83]. Applied to the egg-specific studies synthesized in this review, several of the studies discussed above illustrate these points, and the validation practices of each are summarized here rather than treating every reported accuracy as equivalent. Yimenu and colleagues’ e-nose study used an explicit, independently drawn calibration/validation split (35 and 28 samples, respectively) [39]; Li and colleagues’ e-nose regression models reported both training and testing R^2^ values (0.9641 and 0.8339 for yolk-index prediction), making the generalization gap visible rather than concealing it behind a single figure [41]; and Hassan et al. released a public 186-egg dataset and code and evaluated their models via k-fold cross-validation [56]; as described in that paper, SMOTE oversampling and PCA were applied to the full feature set before the cross-validation folds were evaluated, so the leakage of information between training and test folds cannot be excluded [56]. These are examples of appropriate methodological transparency, not necessarily of strong generalization, since a visible train–test gap is still a gap; the point is that the studies that report both numbers allow a reader to judge that gap directly, while studies reporting only a single top-line accuracy figure do not.

Four validation concerns recur across the egg-AI literature synthesized here. First, dataset size and representativeness: many studies still rely on one flock, one facility, one breed, or a narrow storage protocol. Second, validation hierarchy: a held-out subset from the same acquisition campaign is not equivalent to independent-source validation. The tiers defined in Section 2 form a hierarchy: (1) training performance, (2) internal cross-validation, (3) held-out same-source testing, (4) independent-source validation (new eggs from a different flock or lot, or bought at retail, measured with the same laboratory workflow), (5) cross-facility, cross-season, or geographic testing (changed site, instrument, season, or population), and (6) prospective production-line validation. Third, class imbalance and decision asymmetry: overall accuracy can conceal poor minority-class recall when cracks, contamination, or other safety-critical states are rare. Balanced accuracy, sensitivity/recall, specificity, precision-recall curves, F1-score and, where relevant, calibration should therefore be reported alongside accuracy. Fourth, data leakage: wavelength selection, normalization, feature extraction, augmentation, PCA, SMOTE, or hyperparameter tuning performed before the validation split can transfer information from the test set into model development. The literature often does not report enough detail to determine whether every preprocessing step was nested correctly. These are reporting and design limitations, not proof that an individual model is invalid; they determine how far the reported performance can legitimately be generalized.

None of this should be read as doubt about whether the underlying sensing and AI technologies actually work. The mechanistic basis for most of them is sound. Spectral absorption tracks known chemical changes; image features track visible physical defects. Where methodology is transparent, reported accuracies are frequently high. The point is narrower than a blanket skepticism, and it follows the same evidence-hierarchy logic applied throughout: a high accuracy figure obtained on a limited, single-site laboratory dataset demonstrates proof-of-concept, not industrial robustness. Conflating the two is costly when commercial-scale deployment is considered. Table 4 brings together representative machine-learning, deep-learning, multimodal, and XAI studies discussed in this review, with dataset size and validation practice made explicit. “Not reported” means that the reviewed source did not provide enough information for a confident entry; it does not imply that the procedure was not performed.

The rightmost column should now be read as a validation map rather than a binary claim that “external validation is absent.” Bischof et al. provide the clearest independent-source test in this review (Tier 4), with performance dropping sharply on supermarket samples [28]; because their method needs yolk extraction, the study is included for its validation design and not as an intact-egg technology. More recent studies have strengthened internal rigor: Nukala et al. evaluated three breeds across three storage temperatures and 42 days using leakage-free preprocessing and nested cross-validation, but this still does not constitute cross-facility prospective validation [52]. Liu et al. described their near-infrared hyperspectral model as cross-temporal, but all 300 eggs were obtained on the day of production and followed in one storage experiment (days 1, 20, and 40), and the 20% test set was a random split of that campaign (Tier 3); “cross-temporal” there therefore means across storage time, not across seasons or facilities. Their single-task models lost about 11 percentage points of variety accuracy between day 1 and day 40 (81.2% to 70.4%), whereas the multi-task design held it near 86% [12]. Nakaguchi et al. derived air-cell thresholds and tested sorting on the same 600 quail eggs, which supports Tier 1 only [16]. Pakuła et al. classified eggshells from healthy and Mycoplasma-infected hens with F-scores of about 99%, but healthy eggs came from one reference flock and infected eggs from commercial flocks, and 2,521 measurements (several per shell) were split randomly 7:3, so flock identity and repeated measurements are confounded with the label and only internal validation is supported (Tier 2) [21]. Hassan et al. released a public dataset and code and used cross-validation, improving reproducibility, yet the study remains based on a small, single-dataset development environment [56]. The evidence therefore supports a more precise conclusion: validation practice is improving, but external, prospective, and decision-oriented validation remains sparse. The principal methodological gap is not the absence of validation altogether; it is the scarcity of validation tiers that test whether models remain reliable under real biological and operational domain shift.

## 5. AI-Enabled Quality Prediction and Automated Grading

Section 3 and Section 4 were organized around, respectively, sensing hardware and algorithm families. This section reorganizes the same underlying evidence around the prediction *task* itself, freshness, defects, yolk characteristics, composition, spoilage, and authentication, because a plant manager or a regulator does not select a sensing technology in the abstract; they select whichever technology and algorithm combination best solves a specific quality or safety question. Organizing this way also makes a specific methodological caution easier to enforce: a reported accuracy, R^2^, or F1-score is only meaningful relative to the dataset, class distribution, and validation procedure that produced it, and this section deliberately avoids ranking studies against one another by their headline metrics alone, for exactly the reasons developed in Section 4.4.

### 5.1. Freshness and Haugh Unit Prediction

Freshness prediction, primarily framed around estimating or classifying Haugh unit, is the task with the broadest cross-technology evidence base of any reviewed in this manuscript: it has been approached via hyperspectral imaging [37], near-infrared spectroscopy [33,34], Raman spectroscopy [35,42], fluorescence spectroscopy [36,40], electronic nose sensing [38,39,41], and hyperspectral imaging with band selection and deep networks [54]. Read together, this body of work supports one conclusion with reasonable confidence: freshness-related biochemical change, albumen thinning, pH rise, moisture, and CO_2_ loss leave a detectable signature across a wide range of physical measurement principles, optical, vibrational, and chemical alike. That breadth matters. It suggests that a freshness assessment is not bottlenecked on the unavailability of any single sensing principle, but on the validation and standardization questions raised earlier.

What this body of work does not support is ranking these technologies against one another by reported accuracy figures alone, and that comparison is deliberately not attempted here. The freshness studies reviewed here differ in the outcome type (some predict a continuous Haugh unit value via regression, others classify eggs into discrete freshness or storage-time categories), in dataset size and composition (from tens to several thousand eggs), in storage protocol (constant versus fluctuating temperature, laboratory versus real cold-chain conditions), and in whether training and testing accuracy are reported separately or conflated into one figure. Under these conditions, a Raman study’s classification accuracy and an NIR study’s regression R^2^ are not measuring the same thing closely enough to be ranked on a single scale, and reviews that present such comparisons as though they were commensurable risk suggesting a settled “best” technology where the evidence does not support one.

Where comparison within a single, controlled study is possible, it is considerably more informative than comparison across studies. Kim and colleagues’ cold-chain study is a useful example: predicting the Haugh unit from non-destructively measured weight loss, their transfer-learning CNN-LSTM model reduced the RMSE from 6.62 for a random-forest comparator to 2.02 within the same study and validation framework [81]. More recent work illustrates why storage and population variability must be treated as model inputs rather than nuisance factors. Ong et al. used HSI from 450–1100 nm with distance-correlation wavelength selection and a CNN, obtaining a correlation coefficient of 0.9056 and RMSE of 4.4152 for freshness prediction [53]. Nukala et al. extended the HSI assessment across three breeds and three storage temperatures over 42 days, reporting grading accuracies above 92% at 3 °C, 88% at 20 °C, and 85% at approximately 33 °C, with nested cross-validation and leakage-free preprocessing [52]. These newer studies strengthen the evidence for robustness under controlled variation, but they also show why cross-study headline comparisons remain inappropriate: the targets, storage conditions, preprocessing pipelines, and validation designs are not identical.

### 5.2. Shell and Internal Defect Detection

Defect detection, cracks, pinholes, dirt, blood spots, and (for hatching eggs specifically) infertility, is the task where computer vision and hyperspectral imaging have produced the most industrially oriented results reviewed in this manuscript, and Section 3.1 and Section 3.2 already detailed the leading examples: Yang and colleagues’ 94.8% grading accuracy on a five-category defect classification [25], Chen and colleagues’ 100% defect-inspection accuracy (95% in a 31 ms real-time configuration) from hyperspectral imaging with band selection and CNNs [54], and Li and colleagues’ F1-score of 75.49% for the harder sub-problem of fine crack detection via hyperspectral imaging [31]. These figures span the mid-70s to 100%. The spread should not be read as a ranking of algorithms: the studies differ in the task, dataset, and validation design, and part of it plausibly reflects differences in task difficulty. Gross external defects, dirt, obvious cracks, and size and shape outliers, are comparatively easy targets for any modern computer-vision system, and the grading accuracies reported for them in the studies above exceed 90% [7,25]. Fine eggshell cracks, particularly on pigmented shells or against complex backgrounds, remain harder because the visual signal is subtle, which is why the specialized hyperspectral and attention-based architecture developed by Li and colleagues, built specifically to address this harder case, reports a correspondingly more modest F1-score [31]. Reading these figures side by side without accounting for task difficulty would understate progress on gross defects and overstate how solved fine-crack detection is. Internal defects have so far been tested mostly on induced cases: Al-Mamun et al. injected blood into eggs or ruptured the vitelline membrane to simulate runny yolk (100 induced and 100 control eggs per defect and shell color), and visible–NIR spectroscopy with discriminant analysis classified blood spots at 97.50% (white) and 98.37% (brown) and runny yolk at 99.01% and 100.00% under stratified five-fold cross-validation (Tier 2), whereas a pixel-threshold imaging method detected 92.00% and 88.00% of the blood-spot eggs and could not segment runny yolk; natural defects were not tested [17]. For cracks, near-infrared reflectance sorting of 720 eggs with bump-induced cracks reached 100% (pink and green shells) and 98.75% (white) accuracy on a one-third hold-out set (Tier 3) [19], and an electrical-property method detected artificial microcracks made by a striking machine in 770 eggs with up to 99.44% accuracy, without a described test split [20]. A 2025 review of external eggshell quality likewise lists variation in the shell color, texture, and breed, and the absence of standardized datasets and benchmarks, among the barriers to industrial deployment [23].

### 5.3. Yolk Characteristics

Yolk-focused prediction, principally the yolk index and yolk color/carotenoid content, has been approached through electronic-nose sensing combined with wavelet feature extraction [41] and through fluorescence spectroscopy targeting vitamin A specifically [40]. The comparison between these two bears on a point raised earlier in Section 3.6: yolk vitamin A fluorescence gave 94.9% correct classification in calibration [40], whereas tryptophan fluorescence of thick and thin albumen gave 57.1% in the companion study [36]. Because these results come from different egg fractions, they are not a like-for-like comparison of fluorophores, but they suggest that the choice of target marker can matter as much as the choice of sensing principle.

### 5.4. Chemical Composition

The direct AI-based prediction of egg chemical composition, protein or lipid fractions, fatty acid profile, and specific vitamin concentrations, rather than composition-correlated freshness or authenticity classification, is the least developed prediction task reviewed in this section. Most of the composition-relevant evidence surfaced in this review is indirect: NIR and Raman spectra are sensitive to protein and lipid signatures as a mechanistic basis for the freshness and authentication predictions discussed elsewhere in this section [33,34,35], and the lipidomic and NMR-based authentication studies discussed in Section 5.6 quantify compositional differences between housing systems as a byproduct of their classification task rather than as a standalone compositional prediction goal. A dedicated body of work using AI specifically to predict the quantitative protein or lipid content, analogous to the quantitative shelf-life regression discussed in Section 5.1, was not identified in the material available for this review in comparable depth to the other tasks discussed here, and that absence is noted as a specific gap for future work rather than one filled by extrapolation from the freshness and authentication literature.

### 5.5. Spoilage and Shelf-Life Prediction

Shelf-life prediction extends freshness assessment (Section 5.1) from a single-timepoint estimate into a forward-looking prediction of remaining usable life under specified or variable storage conditions, and it is a task where the storage environment itself, not just the egg, is a necessary model input. Kim and colleagues’ cold-chain study, already discussed in Section 5.1, is directly relevant here because its entire premise is shelf-life-relevant: predicting the Haugh unit trajectory from weight loss under real, fluctuating cold-chain temperature conditions rather than the constant laboratory temperatures more commonly used elsewhere in the freshness literature [81]. That distinction, constant-temperature laboratory storage versus variable real-world cold-chain conditions, matters more for shelf-life prediction specifically than for single-timepoint freshness classification, because a model trained only on constant-temperature data has no basis for extrapolating to the temperature fluctuations that an egg actually experiences among the farm, processor, distributor, and retailer. Separately, hybrid machine-learning models that include laying-hen age as an input have been proposed. In one study, a back-propagation neural network optimized via a cuckoo search and boosted with AdaBoost predicted the remaining shelf life with an RMSE of 0.68 and R^2^ of 0.97 on the test set, and removing hen age lowered the accuracy (R^2^ = 0.95, RMSE = 1.00) [84]. Arrhenius-type kinetic equations remain the classical approach to temperature-dependent quality loss, but no study combining them with machine learning was identified in the material examined.

### 5.6. Authentication

Authentication, principally distinguishing eggs from different housing or husbandry systems (caged, barn, free-range, organic), sits at the intersection of the chemical-composition and food-fraud concerns introduced in Section 2.4, and it has attracted large and methodologically diverse literature: nuclear magnetic resonance spectroscopy of yolk lipids [28], lipidomic mass spectrometry combined with FT-NIR [44], and elemental profiling of the eggshell itself via atomic emission spectrometry [45] have all been used to build classification models distinguishing husbandry systems, generally with high reported laboratory accuracy: Lösel and colleagues reported 96.3% classification accuracy for organic versus conventional eggs using a random forest model on lipidomic and FT-NIR data from 270 eggs [44], and Xie and colleagues reported 95.3% overall accuracy, 95.6% sensitivity, and 95.1% specificity for a least-squares support vector machine distinguishing free-range from caged eggs based on the eggshell elemental profile [45]. These authentication methods are laboratory analytical techniques that need sample extraction or preparation (for example yolk lipid extraction for NMR), so they are not non-destructive assessments of an intact egg. They are discussed because they carry the clearest validation evidence in the authentication literature. Earlier studies on smaller sample sets used UV-Vis-NIR spectroscopy of the yolk lipid extract [29] and egg plasma [30], 1H NMR [85,86], and targeted lipidomics [87]; according to Bischof et al., sample sizes in this earlier literature ranged from 34 to 357 [28].

One study in this sub-literature needs closer attention than a single accuracy figure allows, because it is, among every study synthesized in this review, the clearest and most directly quantified illustration of the internal-versus-external validation gap that this review has emphasized since Section 4.4. Bischof and colleagues built classification models across seven machine-learning algorithms (linear and quadratic discriminant analysis, PLS-DA, support vector machine, random forest, k-nearest neighbors, and an artificial neural network) using ^1^H NMR spectra from 4188 authenticated egg yolks spanning four housing systems, four hen breeds, and the full laying period, the largest authenticated egg-authentication dataset reviewed here [28]. Under standard internal validation, an 80/20 train-test split combined with 10-fold cross-validation, the support vector machine model reached a cross-validation accuracy of 98.5% and a held-out test-set accuracy of 99.0%, the strongest of the seven algorithms compared. The authors then purchased 290 eggs directly from supermarkets and discount retailers, independent samples with a labeled but unverified housing system, and applied all seven trained models to them. Accuracy dropped sharply. The support vector machine model, the strongest performer under internal validation, correctly classified only 52.8% of the supermarket eggs by their labeled housing system; the best-performing model on this external set was in fact linear discriminant analysis at 62.8%, which ranked third under internal cross-validation, and the poorest model (k-nearest neighbors) reached 45.9%. The supermarket set contained only three classes, because colony-cage eggs were not commercially available [28].

**Figure 3:**
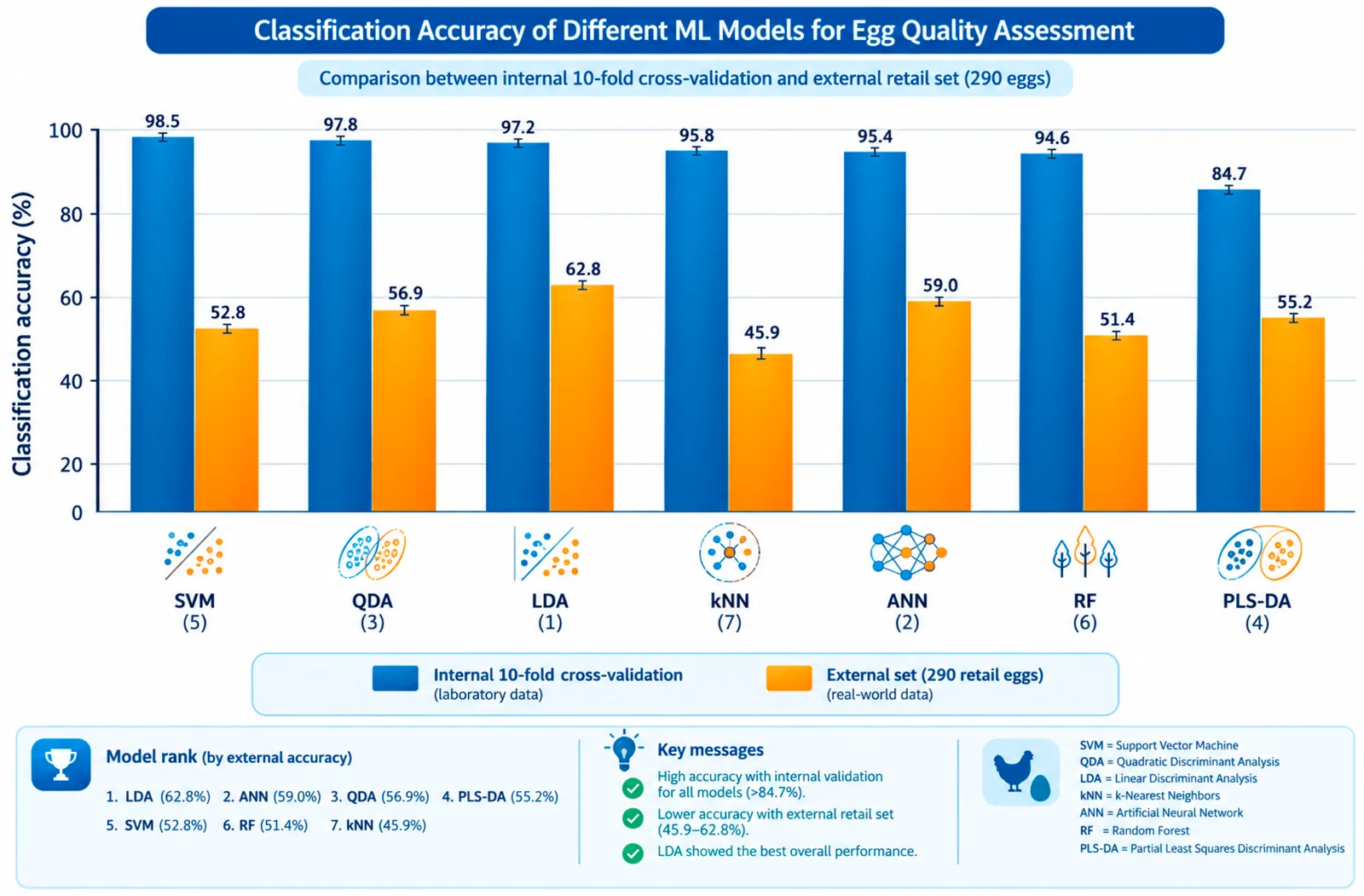
Internal versus external accuracy of seven machine-learning models that authenticate the laying-hen housing system from 1H NMR spectra of egg yolk extracts. Data are taken from Table 1 and Table 2 of Bischof et al. [28] (open access, CC BY 4.0). Blue bars show 10-fold cross-validation accuracy on the 4188-egg development set. Orange bars show accuracy on 290 eggs bought from supermarkets and discount retailers and classified against the housing system printed on the label; these labels were not independently verified, and colony-cage eggs were not available, so the external set has three classes. Models are ordered by internal accuracy. The dashed line marks 98.5%, the best internal cross-validation accuracy (SVM). External accuracy was 35.7 to 52.6 percentage points below that value. LDA, third by internal accuracy, had the highest external accuracy (62.8%); the SVM, first internally, ranked fifth of seven externally (52.8%).

The drop should not be read simply as a failure to generalize. The authors offer two explanations. Part of the gap may reflect mislabeling in the commercial supply chain, itself a food-fraud finding relevant to the authentication problem that the study set out to address, and part reflects real biological variability (hen breed, feeding regime, and a former rule that eggs from free-range hens kept indoors for more than 12 weeks had to be labeled as barn eggs, which changed in November 2023) that the training set, despite being unusually large, did not fully sample [28]. Both explanations point to the same underlying methodological lesson already established in Section 4.4: a model’s cross-validation accuracy describes how well it fits the statistical structure of its own training population and says comparatively little about performance against the full range of variability, biological, seasonal, and supply-chain, that an independent, real-world sample introduces. That LDA, which ranked third via internal cross-validation (97.2%), had the highest external accuracy, while the SVM, which ranked first (98.5%), ranked fifth of seven, is a stronger caution than a uniform drop would have been: internal validation metrics do not necessarily rank models correctly for real-world performance, let alone estimate absolute accuracy. This discrepancy between internal validation and independent external performance is illustrated in Figure 3. No other egg-AI study synthesized in this review tests this directly, which is why Table 4 assigns Tier 4 only to Bischof and colleagues’ study, the one exception in the literature surveyed here. The result is best read as an example of a risk that most of the literature has not tested, not as an isolated anomaly.

## Figures and Tables

**Table 1:** Positioning of this review relative to major recent reviews, with the present contribution centered on validation, generalization, and sensing-to-decision integration.

Review	Year	Journal	Scope	Main Contribution	Main Limitation (Relative to This Review’s Aim)	What This Review Adds
Ahmed et al. [7]	2023	Compr Rev Food Sci Food Saf	Non-destructive optical sensing across the egg industry, framed around “Industry 4.0”	Broadest existing synthesis of optical sensing modalities and their industrial-readiness barriers	Processing (thermal or non-thermal) is outside its scope; sensing is not connected to a process-selection or optimization step	Traces sensing output through to a non-thermal process-selection and functional-preservation decision, which [7] does not attempt
Ahmed et al. [11]	2025	Comput Electron Agric	Hyperspectral imaging specifically, in the egg and hatchery sector	Focused, up-to-date synthesis of one sensing modality’s egg and hatchery applications	Single-modality scope by design; hatchery/embryo applications sit outside food-processing relevance; no processing linkage	Treats HSI as one of several complementary modalities within a multimodal architecture and restricts scope to food-processing-relevant applications only
Atwa et al. [8]	2024	Foods	Emerging non-destructive technologies for raw egg freshness detection	Wide-ranging survey of freshness-detection modalities including less common techniques (fluorescence, thermal imaging)	Detection and characterization only; does not address what happens to an egg once freshness or safety status is established	Extends the pathway from a freshness/safety prediction into an explicit process-selection and preservation-verification step
Gao et al. [10]	2025	Foods	Comprehensive evaluation methods for egg freshness (sensory, physical, chemical, intelligent)	Integrates traditional and intelligent freshness-evaluation methods within one framework	Framework stops at evaluation; does not connect freshness status to a processing or preservation decision	Adds the AI-to-processing linkage and the non-thermal preservation question this framework does not address
Yang et al. [9]	2025	Compr Rev Food Sci Food Saf	Freshness and safety detection across both shell eggs and liquid egg products	Most current and broadest freshness/safety detection synthesis; also extends scope to liquid egg, where evaluation standards are largely absent	Detection-focused; liquid egg’s expanded scope is not connected to the non-thermal processing decisions liquid egg products are most relevant to	Connects the liquid-egg detection gap this review identifies to the liquid-egg non-thermal processing literature synthesized in Part II
Bermudez-Aguirre & Niemira [6]	2023	Compr Rev Food Sci Food Saf	Traditional and novel (non-thermal) egg pasteurization and disinfection technologies	Authoritative synthesis of microbial-inactivation mechanisms and quality trade-offs across thermal and non-thermal methods	Does not engage with AI-based sensing, prediction, or any data-driven process-control layer	Adds the sensing-and-AI layer that could inform which of the technologies this review surveys gets applied to which batch and with what intensity
Liu et al. [5]	2019	Compr Rev Food Sci Food Saf	Effects of novel processing techniques on the functional properties of egg proteins	Detailed mechanistic treatment of how specific processing techniques alter foaming, gelling, and emulsifying properties	Functional-property focus only; not connected to sensing, prediction, or process automation	Frames functional-property preservation as one design objective within a larger sensing-to-processing-to-verification system, rather than as a standalone processing question

**Table 2:** Egg quality and safety attributes relevant to smart processing, the sensing technologies reported to access them, and whether current evidence supports direct measurement or indirect prediction. Authentication entries (NMR, lipidomic mass spectrometry, eggshell elemental profiling) and biosensor entries rely on sample extraction or preparation. They are laboratory analytical methods, not non-destructive measurements of an intact egg.

Attribute	Category	Conventional (Destructive) Method	Non-Destructive Sensing Technology Reported	Measured Directly or Predicted	Readiness Level Reported (Not a Validation Tier)
Shell cracks/pinholes	External	Visual/manual inspection, dye penetration	Computer vision [7,25] (Section 3.1); hyperspectral imaging [31] (Section 3.2)	Measured (physical defect visible or spectrally resolvable)	Industrial (vision); laboratory (HSI fine cracks)
Shell color, shape, size	External	Visual grading, calipers	Computer vision [25,32] (Section 3.1)	Measured directly	Industrial
Surface contamination/dirt	External	Visual inspection	Computer vision [32] (Section 3.1)	Measured directly	Industrial
Haugh unit/albumen height	Internal	Break-out, tripod micrometer	NIR [33,34] (Section 3.4), Raman [35] (Section 3.5), fluorescence [36] (Section 3.6), HSI [37] (Section 3.2), e-nose [38,39] (Section 3.7)	Predicted (spectral/chemical correlate of albumen biochemistry)	Laboratory
Yolk index/yolk color	Internal	Break-out measurement	Fluorescence spectroscopy targeting vitamin A [40] (Section 3.6); e-nose [41] (Section 3.7)	Predicted	Laboratory
Air cell height	Internal	Candling (semi-destructive), break-out	NIR spectroscopy [34] (Section 3.4); microwave spectroscopy [14,15] and thermal imaging [16] (Section 3.9)	Predicted	Laboratory
Internal defects (blood/meat spots, scattered yolk)	Internal	Candling, break-out	Computer vision candling; HSI [37] (Section 3.2); Vis-NIR spectroscopy [17] (Section 5.2)	Measured (visible) or predicted (subtle cases)	Laboratory–Industrial (candling)
Protein composition (ovalbumin, ovotransferrin)	Chemical/nutritional	Wet chemistry, electrophoresis	NIR, Raman spectroscopy [33,35] (Section 3.4 and Section 3.5); UV–Vis transmittance (S-ovalbumin) [13] (Section 3.4)	Predicted	Laboratory
Lipid content, fatty acid profile	Chemical/nutritional	Solvent extraction, GC	NIR, Raman spectroscopy; NMR-based authentication methods [28] (Section 5.6)	Predicted	Laboratory
Carotenoids/fat-soluble vitamins	Chemical/nutritional	HPLC	Fluorescence spectroscopy [40] (Section 3.6; Raman [42] (Section 3.5)	Predicted	Laboratory
Moisture, pH	Chemical/nutritional	Direct titration/probe (destructive)	NIR spectroscopy [33] (Section 3.4)	Predicted	Laboratory
Microbial contamination (*Salmonella*)	Safety	Culture-based testing	Biosensors [43] (Section 3.8; sample-based assays)	Measured directly (biosensor) or predicted (all other modalities, via spoilage proxy)	Laboratory (biosensors); Not applicable (indirect modalities cannot substitute)
Spoilage/volatile compounds	Safety	Sensory panel, TVB-N assay	Electronic nose [38,39,41] (Section 3.7)	Predicted (indirect chemical correlate)	Laboratory
Authenticity/production-system origin	Safety/authenticity	Certification and paper-trail audit	NMR [28] (Section 5.6), lipidomic MS + FT-NIR [44] (Section 5.6), eggshell elemental profiling [45] (Section 5.6)	Predicted (statistical classification)	Laboratory, with one study showing severe internal-to-external accuracy degradation [28]

**Table 3:** Advanced sensing technologies applied to eggs: principle, target attributes, speed, relative cost, industrial readiness, major limitation, and representative evidence. Values in the last column come from different tasks, datasets and metrics and are not comparable across rows; “not extracted” means the item was not collected for this review.

Technology	Principle	Primary Target Attributes	Speed	Relative Cost	Industrial Readiness	Major Limitation	Representative Evidence (Sample Size; Performance; Highest Tier)
Computer vision (Section 3.1)	RGB/grayscale imaging, classical or CNN-based classification	External defects, shape, size, grading	Sub-millisecond to low millisecond per image [7]	Low (commodity hardware)	Industrially established for grading/sorting [32]; laboratory/pilot-scale for combined grading-and-weighing [25]	Surface-limited; cannot access internal or chemical attributes	Compared in [7,50]: mean precision 94.0%, 91.25%, and 85.75% for normal, dirty, and cracked eggs (primary study [50]; 70 test images). RTMDet + RF [25]: 94.8%, R^2^ = 0.960; sample size not reported. Tier not determinable.
Hyperspectral imaging (Section 3.1)	Spatially resolved reflectance/transmittance across a wide spectral range	Freshness, scattered yolk, fine shell cracks	Seconds per acquisition; large data cubes	High (research-grade cameras)	Emerging–pilot ready [31,37,51,52,53]	Egg geometry (curvature, specular reflection); shell-pigmentation confound; data volume	Ref. [31]: 400 eggs (self-constructed), F1 = 75.49%, tier not determinable. Ref. [52]: 1500 eggs, 3 breeds, 3 temperatures, >85–92% grading accuracy, Tier 2 (nested CV).
Multispectral imaging (Section 3.3)	Reduced-waveband imaging, often derived from HSI wavelength selection	Freshness, defects	Comparable to computer vision once bands reduced	Moderate	Emerging–pilot ready [54]	Dedicated purpose-built hardware still uncommon; mostly a reduced form of HSI	Ref. [54]: HSI with band selection and CNNs; 99% (freshness), 100% (defect inspection), 95% at 31 ms; 1800 eggs in a secondary summary [11], not checked in [54]; tier not determinable.
Near-infrared spectroscopy (Section 3.4)	Point absorption spectroscopy, through-shell	Freshness (Haugh unit, albumen height), composition	Seconds per reading	Low–moderate (portable units available)	Developing–pilot ready [33,34]	No spatial resolution; cannot localize a defect	Sample sizes, metrics, and tiers of [33,34] were not extracted for this review.
Raman spectroscopy (Section 3.5)	Vibrational (inelastic) light scattering	Freshness, authentication	Seconds per reading	Moderate–high	Emerging (laboratory) [35,42]	Fluorescence background interference from egg pigments	Ref. [42]: sample size not reported; 80% (validation set); tier not determinable.
Fluorescence spectroscopy (Section 3.6)	Native fluorophore emission (tryptophan, vitamin A, porphyrins)	Freshness (yolk-specific), cuticle condition	Seconds per reading	Moderate	Emerging (laboratory) [36,40]	Performance highly fluorophore-dependent (Section 3.6)	Refs. [36,40]: 57.1%/51.9% (albumen, tryptophan) and 94.9%/91.4% (yolk, vitamin A), calibration/validation; sample sizes not extracted; one study population.
Electronic nose (Section 3.7)	Gas-sensor array or fast-GC volatile profiling	Freshness, storage time, spoilage	Minutes (headspace equilibration)	Low–moderate	Emerging (laboratory) [38,39,41]	Slow relative to imaging; sensor drift; humidity/temperature cross-sensitivity	Ref. [39]: 35 calibration + 28 validation eggs. Ref. [41]: 92.86% (test); yolk-index R^2^ = 0.9641 (train) vs. 0.8339 (test). Tier 3.
Biosensors (Section 3.8)	Antibody/aptamer/phage/DNA recognition + electrochemical, optical, or piezoelectric transduction	Pathogen detection in swab, rinse or homogenate samples; not intact-egg assessment (principally *Salmonella*)	Minutes to hours depending on assay	Moderate–high	Laboratory proof-of-concept only [43]	Validated against spiked samples only; not tested against real matrix/contamination complexity	Spiked samples only [43]; LOD 1.6 × 10^2^ CFU/cm^2^ (magnetoelastic sensor, eggshell); no real-matrix validation.
Other emerging technologies (Section 3.9): thermal imaging, LF-NMR, terahertz, dielectric, ultrasound	Various (thermal emission, water-proton relaxation, sub-mm wave, dielectric permittivity, acoustic)	Freshness, internal composition	Varies	Varies	Emerging; evidence depth varies by modality [8,10]	Evidence base too thin to characterize reliably at this stage	Too few comparable studies to tabulate. Single-site studies of microwave spectroscopy [14,15], thermal imaging [16] and computed tomography [22] are listed in Table 4.

## Data Availability

No new data were created or analyzed in this study. Data sharing is not applicable to this article.

## Supplementary Materials

The following supporting information can be downloaded at: https://www.mdpi.com/article/10.3390/foods15213956/s1. Table S1: Supplementary search log (23–24 October 2026, general web search engine); Table S2: Reference audit (87 references); Table S3: Evidence tiers for Table 4 studies; Table S4: Claim-to-source matrix for key quantitative claims; Table S5: Response status matrix (35 comments); Table S6: Database search flow and screening log (81 unique records).
